# Covalent Inhibition of the Histamine H_3_ Receptor

**DOI:** 10.3390/molecules24244541

**Published:** 2019-12-11

**Authors:** Gábor Wágner, Tamara A. M. Mocking, Albert J. Kooistra, Inna Slynko, Péter Ábrányi-Balogh, György M. Keserű, Maikel Wijtmans, Henry F. Vischer, Iwan J. P. de Esch, Rob Leurs

**Affiliations:** 1 Amsterdam Institute for Molecules, Medicines and Systems (AIMMS), Division of Medicinal Chemistry, Faculty of Science, Vrije Universiteit Amsterdam, De Boelelaan 1108, 1081 HZ Amsterdam, The Netherlands; gaborwagner75@gmail.com (G.W.); t.a.m.mocking@vu.nl (T.A.M.M.); innaslynko@gmail.com (I.S.); m.wijtmans@vu.nl (M.W.); h.f.vischer@vu.nl (H.F.V.); i.de.esch@vu.nl (I.J.P.d.E.); 2 Medicinal Chemistry Research Group, Research Centre for Natural Sciences, Hungarian Academy of Sciences, Magyar tudósok krt 2, H-1117 Budapest, Hungary; abranyi-balogh.peter@ttk.mta.hu (P.Á.-B.); keseru.gyorgy@ttk.mta.hu (G.M.K.)

**Keywords:** G protein-coupled receptor (GPCR), Histamine H_3_ receptor, covalent binder, isothiocyanate

## Abstract

Covalent binding of G protein-coupled receptors by small molecules is a useful approach for better understanding of the structure and function of these proteins. We designed, synthesized and characterized a series of 6 potential covalent ligands for the histamine H_3_ receptor (H_3_R). Starting from a 2-amino-pyrimidine scaffold, optimization of anchor moiety and warhead followed by fine-tuning of the required reactivity via scaffold hopping resulted in the isothiocyanate H_3_R ligand **44**. It shows high reactivity toward glutathione combined with appropriate stability in water and reacts selectively with the cysteine sidechain in a model nonapeptide equipped with nucleophilic residues. The covalent interaction of **44** with H_3_R was validated with washout experiments and leads to inverse agonism on H_3_R. Irreversible binder **44** (VUF15662) may serve as a useful tool compound to stabilize the inactive H_3_R conformation and to study the consequences of prolonged inhibition of the H_3_R.

## 1. Introduction

The histamine H_3_ receptor (H_3_R) was discovered in 1983 by Arrang et al. [1] as the third representative of the G protein-coupled receptor (GPCR) subfamily of histamine receptors (now known to consist of H_1_R, H_2_R, H_3_R, and H_4_R). As an autoreceptor, H_3_R presynaptically modulates the synaptic histamine level, while as a heteroreceptor it regulates the levels of other neurotransmitters (e.g., acetylcholine, noradrenaline, serotonin, dopamine, glutamate). Due to the high expression in brain areas such as the cerebral cortex, hypothalamus tuberomammillary nucleus, hippocampus, and striatum, H_3_R regulates several physiological processes of the central nervous system including sleep-wake cycle, learning and memory processes, food intake, and susceptibility to seizures [2,3,4]. As such, H_3_R attracts considerable interest as a therapeutic target from industry and academia alike. The cloning of the human receptor in 1999 by Lovenberg et al. [5] and the discovery of several structurally diverse drug-like ligands in the 2000s [6,7] gave a strong impetus to H_3_R research. Although this boom resulted in a large amount of novel H_3_R ligands, several clinical trials ongoing in the early 2010s [8,9] have so far resulted in only one approved H_3_R ligand (Wakix, approved in 2016 by EMA and in 2019 by FDA) [10,11] suggesting that H_3_R pharmacology is not yet fully understood and complementary approaches are needed to pave the way forward.

One such approach is the use of covalent small-molecule binders, which can for example help in the stabilization of H_3_R in the active or inactive conformation. This in turn could aid in crystallization of a ligand-receptor complex (no crystal structure of H_3_R has been published to date), as well in the exploration of the binding site, conformation, and signaling properties of the protein. Targeted covalent inhibitors (TCIs) are ligands designed to bind irreversibly to a nucleophilic amino acid residue when bound to the targeted protein. A typical strategy of TCI design is linking a pharmacophore moiety (anchor) that leads the ligand into the binding pocket to a reactive moiety (warhead) which reacts with a residue creating a covalent bond [12,13]. In the field of kinases the use of TCIs has resulted in marketed drugs in past years [12], while the progress in covalent GPCR ligands is still limited in comparison [13]. Nonetheless, aided by advances in GPCR crystallography, covalent ligands targeting the orthosteric binding site of GPCRs have been disclosed [14,15,16,17,18,19,20,21,22,23]. The targeted GPCR residue is mostly a cysteine with diverse warheads being used, such as a disulfide [17,19,23], isothiocyanate [18], or Michael acceptor [15]. Other residues have been targeted as well with various warheads, i.e., lysine with activated ester [16] or arylsulfonyl fluoride [20], tyrosine with an arylsulfonyl fluoride [22], or asparagine with an aziridinium ion [17]. Indeed, recent extensions of the diversity in warheads give an opportunity for the fine-tuning of the reactivity [24,25].

**Figure 1:**
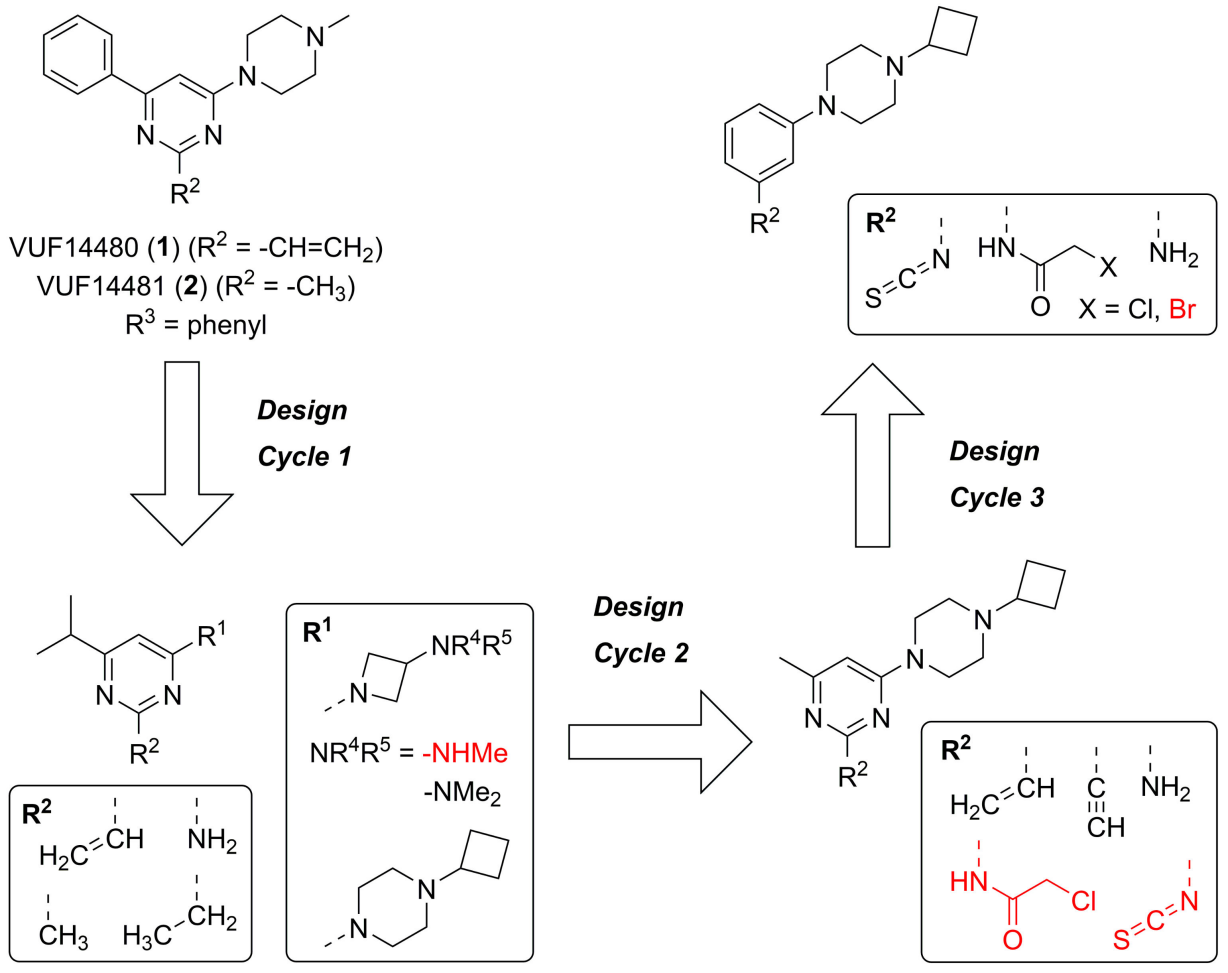
The three design cycles of the potential H_3_R covalent ligands and their non-covalent reference compounds. Red compounds proved too unstable to be isolated in pure form.

While no covalent ligand for H_3_R has been reported to date, the use of covalent strategies within the histamine receptor family is not without precedent. In the 1940s, covalent histamine-protein complexes were explored for pharmacological investigation using different warheads on histamine [26,27]. More recently, mutated H_1_R has been targeted by a disulfide-containing ligand [17]. Furthermore, our group has reported the discovery of a H_4_R covalent ligand VUF14480 (**1**, Figure 1) which acts as a partial H_4_R agonist [15]. Its submicromolar affinity (pK_i_ = 6.3) for human H_4_R is high enough to ensure sufficient recognition by the receptor, whereupon the ethenyl moiety acts as a Michael acceptor forming a covalent interaction with C98^3.36^ [15].

In this study, we describe the design and synthesis of covalent H_3_R ligands, ultimately resulting in the discovery of a covalent inverse agonist targeting the H_3_R.

## 2. Results

### 2.1. Design

**Figure 2:**
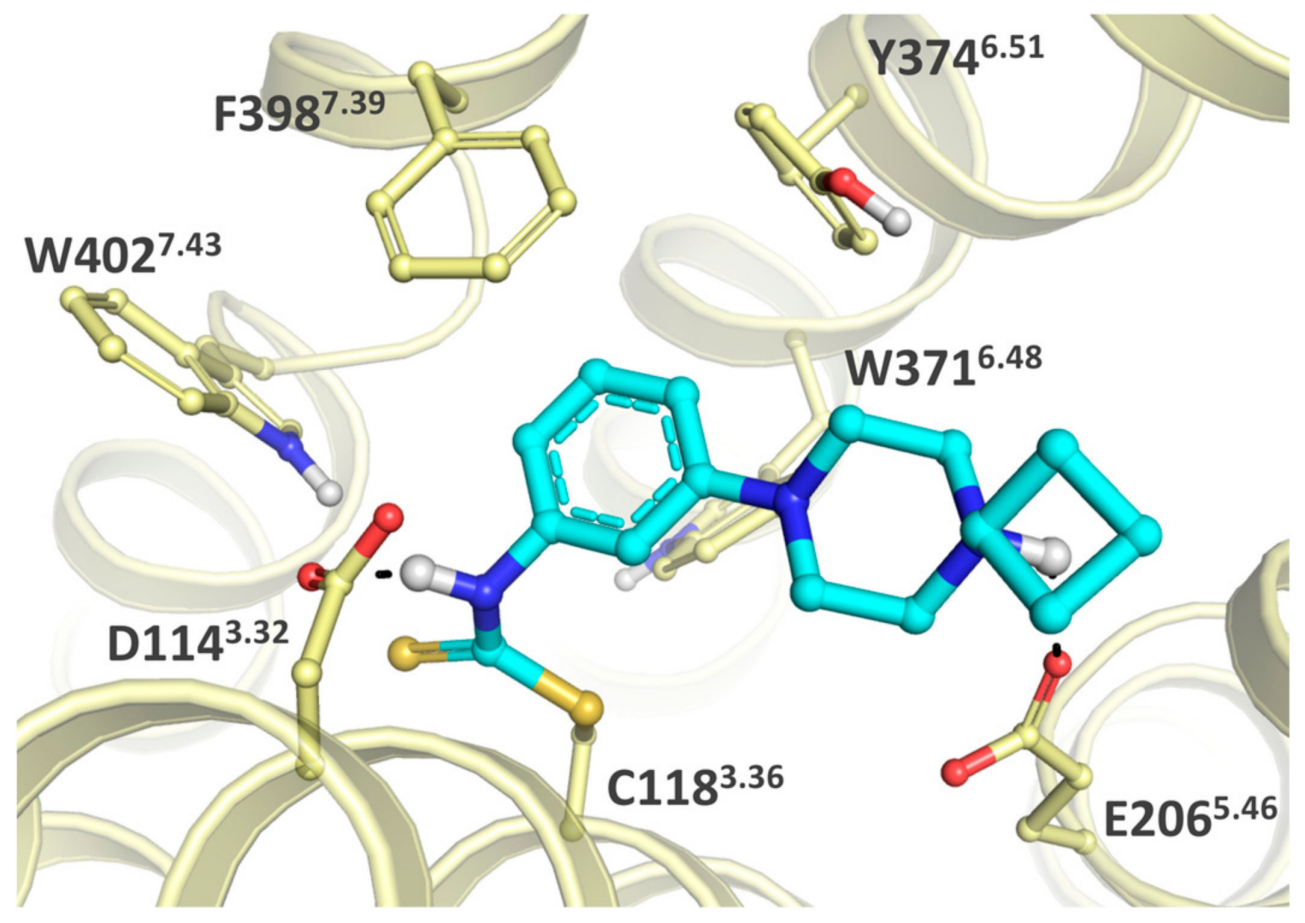
Proposed binding mode of a representative compound from the third design cycle (Figure 1 top right, R^2^ = -NCS) covalently bound to C118^3.36^. The ligand is shown in cyan sticks and the protein is shown in light yellow sticks and ribbons. Only polar hydrogens are shown in white with protein-ligand hydrogen bonding indicated in black dashed lines. For clarity purposes, parts of the protein are not shown. Residue numbers are shown as UniProt and as GPCR-specific Ballesteros–Weinstein numbers [30].

Arguably, a H_4_R covalent ligand can be a suitable starting point for covalent H_3_R ligands given the high homology of H_4_R and H_3_R (43% full sequence identity, 50% predicted transmembrane bundle identity) [28]. Hence, the covalent interaction of Michael acceptor VUF14480 (**1**) with H_4_R [15] was used as a template for the design of potential H_3_R covalent ligands (Figure 1). Similarly to the H_4_R binding pocket, C^3.36^ is also accessible in the H_3_R binding pocket (C118^3.36^) to potentially serve as nucleophilic moiety [29] (throughout this manuscript, we use both the UniProt residue numbers and the GPCR-specific Ballesteros–Weinstein residue numbering scheme [30]). Given the relatively high similarity of the H_3_R and H_4_R orthosteric binding pockets [29], the C118^3.36^ residue of H_3_R might be targeted with a similar combination of anchor, central core and warhead moieties as **1** contains. Several design cycles were needed to tune this scaffold into a H_3_R covalent ligand suitable for our purposes. In-house results (unpublished data) suggested that H_4_R/H_3_R selectivity of this scaffold could be switched from H_4_R to H_3_R by suitable modification of the anchor and substituent pattern of pyrimidine core. Thus, the aim of the first design cycle was the identification of a suitable anchor (R^1^). Three different anchors were chosen based on publicly available and in-house data: (1) replacement of the methyl substituent by a cyclobutyl group [31], (2) replacement of the piperazine moiety by a 3-(methylamino)azetidine moiety, (3) replacement of the piperazine moiety by a 3-(dimethylamino)azetidine moiety to assist in synthetic feasibility (vide infra). Based on in-house results, the phenyl group at Position 6 was changed to an isopropyl group to favor H_3_R selectivity. Both the pyrimidine ring as central core and the vinyl group as warhead were unchanged. Instead, the warhead (R^2^) was addressed in the second design cycle whilst the group at Position 6 was changed to a methyl group to prevent the more lipophilic isopropyl moiety from targeting a lipophilic subpocket next to the cysteine, thereby preventing the warhead from achieving its appropriate orientation. The vinyl unit was changed to an ethynyl moiety in order to potentially improve the orientation of the reactive carbon atom. Isothiocyanate and halomethylamido derivatives were also designed. Notably, isothiocyanates are known to have high reactivity toward cysteines and reduced albeit still substantial reactivity toward lysines [13,32,33] depending on the pH, while chloroacetamides show reactivity only to cysteines [13]. Yet in our work, both warhead types caused synthetic obstacles (vide infra) because of too high chemical reactivity that we attribute to the electron-withdrawing pyrimidine nitrogen atoms. This reactivity issue combined with biological inactivity of other compounds in this second cycle (vide infra) necessitated a third design cycle comprising of scaffold hopping to the less electron-withdrawing phenyl ring as central core. This switch in core was supported by a ChEMBL search on H_3_R ligands containing a phenyl group connected with a 3-carbon linker to a basic amino moiety. Figure 2 illustrates the envisioned strategy key to the third design cycle, with C118^3.36^ forming a covalent bond with the isothiocyanate moiety. The binding mode of a representative ligand from this design cycle (Figure 1 top right, R^2^ = -NCS) was evaluated by covalent docking using GOLD (version 5.2.1). The binding site of the H_3_R protein is characterized by two acidic residues that are known to interact with basic groups of the ligands, i.e., D114^3.32^ and E206^5.46^. Residue D114^3.32^ is considered the anchoring point for the basic amines of the endogenous ligands of aminergic GPCRs [34]. Residues in TM5 are also involved in ligand binding and in the case of H_3_R it is residue E206^5.46^ that is anticipated to interact with the imidazole ring of the endogenous agonist histamine [35]. Interestingly, the predicted binding mode of the isothiocyanate analog is flipped to allow covalent bond formation between the isothiocyanate and C118^3.36^, resulting in a binding mode in which the basic piperazine moiety interacts with E206^5.46^ through an ionic interaction. The nitrogen atom of the dithiocarbamate that is formed after covalent bond formation is able to interact with D114^3.32^ (Figure 2) through a hydrogen bond.

When studying ligands that form covalent bonds with protein targets, it is essential for pharmacological characterization to have proper non-reactive reference compounds. In our previous work on H_4_R ligand **1**, the 2-methyl-analog **2** had served as non-covalent control [15]. Based on steric and electronic considerations, in the current work several 2-methyl, 2-ethyl and 2-amino-derivatives of the designed compound set were investigated as controls.

### 2.2. Synthesis

**Scheme 1:**
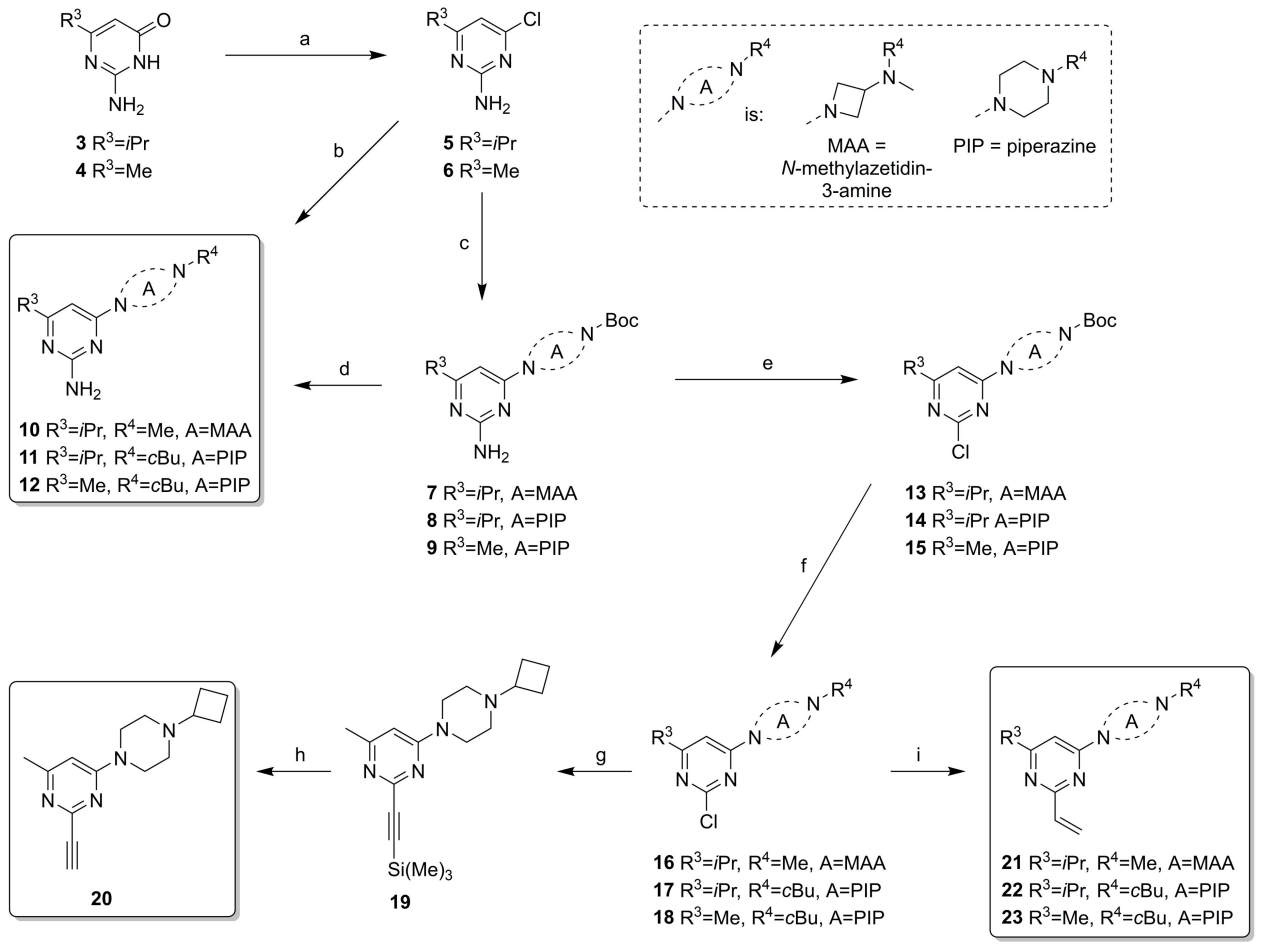
Synthesis of pyrimidine-based covalent ligands. (**a**) POCl_3_, reflux, 3 h, 26–33%; (**b**) *N,N*-dimethylazetidin-3-amine or 1-cyclobutylpiperazine, *N,N*-Diisopropylethylamine (DIPEA), dioxane, 150 °C, 30 min, μW, 59–65%; (**c**) *t*-butyl azetidin-3-yl(methyl)carbamate or *t*-butyl piperazine-1-carbamate, DIPEA, dioxane, 150 °C, 30 min, μW, 50–85%, **8**: used without full purification; (**d**) 1. HCl, dioxane, rt, 2 h, 2. cyclobutanone, NaBH(OAc)_3_, dichloromethane (DCM), rt, overnight, 33% (two steps); (**e**) *t*BuONO or *i*PeONO, SbCl_3_, DCM, rt, 3 h, 23–40%; (**f**) 1. HCl, dioxane, rt, 2 h-overnight, 2. H_2_CO or cyclobutanone, NaBH(OAc)_3_, DCM, rt, overnight, 56–73% (two steps); (**g**) ethynyltrimethylsilane, CuI, Pd(dppf)Cl_2_, TEA, DME, 100 °C, 1 h, μW, 54%; (**h**) K_2_CO_3_, MeOH, rt, 1 h, 72%; (**i**) vinyl boronic acid MIDA ester, Pd(PPh_3_)_4_, Na_2_CO_3_, DME, H_2_O, 120 °C, 1 h, μW, 21–62%.

**Scheme 2:**
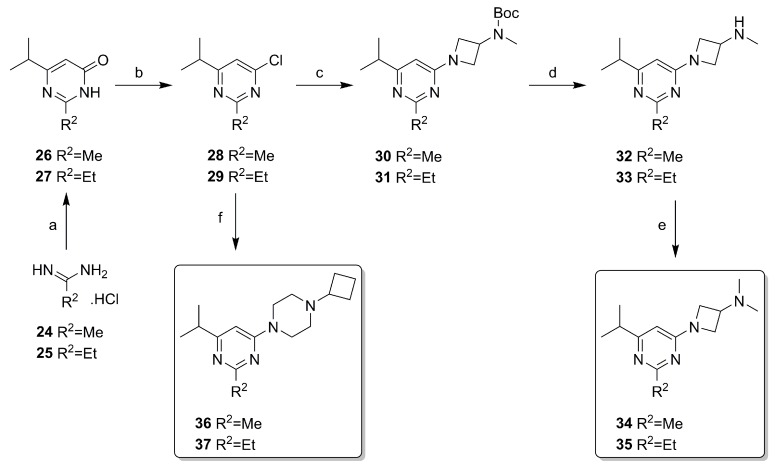
Synthesis of pyrimidine-based reference ligands. (**a**) methyl 4-methyl-3-oxopentanoate, NaOMe, MeOH, rt, overnight, 91–97%; (**b**) POCl_3_, reflux, 3 h, 86–87%; (**c**) *t*-butyl azetidin-3-yl(methyl)carbamate, DIPEA, dioxane, 150 °C, 30 min, μW, 34–38%; (**d**) HCl, dioxane, rt, 3 h-overnight, 77–90%; (**e**) H_2_CO, NaBH(OAc)_3_, DCM, rt, overnight, 82%; (**f**) 1-cyclobutylpiperazine, DIPEA, dioxane, 150 °C, 30 min, μW, 36–51%.

The compounds from Design Cycles 1 and 2 were synthesized as outlined in Scheme 1 and Scheme 2. The core intermediates **5** and **6** were obtained from the appropriate pyrimidin-4(3*H*)-one derivatives **3** or **4** with POCl_3_. The Boc-protected intermediates **7**–**9** were obtained via nucleophilic aromatic substitution on **5** or **6** with aminoazetidine or piperazine moieties under microwave heating at 150 °C. Two 2-amino derivatives (**10** and **11**) were directly synthesized from *N*,*N*-dimethylazetidin-3-amine or 1-cyclobutylpiperazine via an aromatic substitution on **5** or **6**, while the other amino derivative **12** was synthesized from the Boc-protected intermediate **9** via deprotection under acidic conditions followed by a reductive amination. Pyrimidines **7**–**9** were transformed to 2-chloro-pyrimidine intermediates **13**–**15** by nonaqueous diazotization with *t*BuONO or *i*PeONO and SbCl_3_ [36]. Boc-protection was necessary for this step to prevent by-products. Formation of chlorides **13**–**15** was followed by deprotection under acidic condition and reductive amination with H_2_CO or cyclobutanone to afford the intermediates **16**–**18**. A silyl-protected ethynyl group was incorporated via a Sonogashira coupling on intermediate **18** to give **19**, followed by removal of the protective group to yield ligand **20**. The vinyl derivatives **21**–**23** were obtained via Suzuki-Miyaura cross-coupling with vinyl boronic acid N-methyliminodiacetic acid (MIDA) ester on the 2-chloro-pyrimidines **16**–**18** in a microwave at 120 °C. The synthesis of the 3-(methylamino)azetidine analog of **21** (i.e., with R^4^ = H) was undertaken (not shown), although we anticipated a modest stability because of the simultaneous presence of nucleophilic and electrophilic moieties in the product. Therefore, we designed a protocol in which deprotection on a precursor having R^4^ = Boc was carried out in a hot D_2_O/d6-DMSO mixture [37] with the intent to use the solution as is for pharmacological purposes. However, even though product was formed (typically ca. 70% conversion), overall conversion and impurity profiles were not acceptable for further pursuit.

Non-covalent 2-alkyl-pyrimidines were synthesized with similar synthetic steps, as outlined in Scheme 2. Condensation of the appropriate amidines **24** or **25** and 4-methyl-3-oxopentanoate to **26** and **27** was followed by conversion to chloro-analog **28** and **29** with POCl_3_. A nucleophilic aromatic substitution on **28** or **29** with *t*-butyl azetidin-3-yl(methyl)carbamate was performed in a microwave at 150 °C and the obtained Boc-protected intermediate **30** and **31** were deprotected resulting in the monomethyl intermediates **32** and **33**. These in turn were subjected to a reductive amination with H_2_CO to afford **34** and **35**. Likewise, cyclobutyl-piperazine products **36** and **37** were obtained from **28** or **29** and 1-cyclobutylpiperazine.

**Scheme 3:**
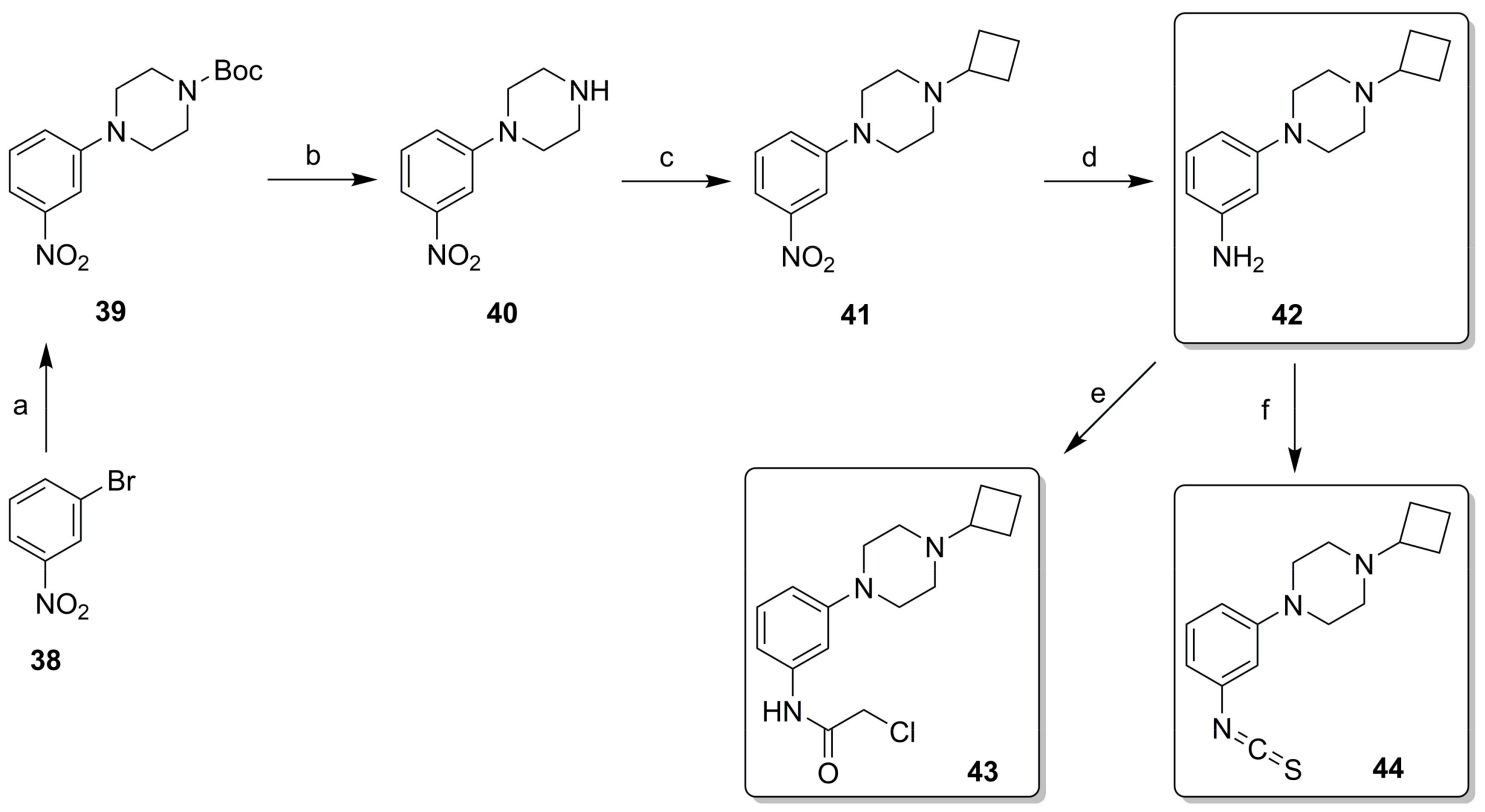
Synthetic of phenylpiperazine derivatives. (**a**) *t*-butyl piperazine-1-carboxylate, NaO*t*Bu, Pd_2_(dba)_3_, Xantphos, dioxane, 80 °C, 1 h, μW, 57%; (**b**) HCl, dioxane, rt, 1 h, 78%; (**c**) cyclobutanone, NaBH(OAc)_3_, DCM, rt, overnight, 76%; (**d**) HCOONH_4_, Pd/C, MeOH, H_2_O, rt, overnight, 25%; (**e**) 2-chloroacetyl chloride, TEA, DCM, rt, 1 h, 68%; (**f**) CSCl_2_, NaHCO_3_, DCM, H_2_O, rt, 1 h, 64%.

In Design Cycle 2, we also attempted to prepare the designed isothiocyanate and chloroacetamide derivatives from compound **12** via reaction with CSCl_2_ or 2-chloroacetylchloride, but all attempts were met by complex product profiles. We attribute this to the too high chemical reactivity of the warhead (vide supra), leading to decomposition during workup and purification. All derivatives with phenyl as core ring (i.e., those from the third design cycle) were synthesized as outlined in Scheme 3. Compound **39** was built via a Buchwald–Hartwig amination of bromide **38** with *t*-butyl piperazine-1-carboxylate. Deprotection to **40** and reductive amination with cyclobutanone yielded **41**. The nitro group of **41** was reduced with HCOONH_4_ and Pd/C to afford key intermediate **42**, which was used for incorporating reactive warheads under basic conditions. Chloroacetamide **43** was formed with 2-chloroacetyl chloride, while isothiocyanate **44** was formed with CSCl_2_. Synthesis of the bromo analog of **43** was also attempted under similar conditions, but the product decomposed during purification. All potential covalent ligands (**20**–**23**, **43**, **44**) were stored as solids in the freezer (−20 °C). Under these conditions, key compound **44** was stable for >2 years (as judged by NMR and LC-MS analysis).

### 2.3. Structure-Activity Relationship

Compounds were evaluated for H_3_R binding in a [^3^H]N-α-methylhistamine ([^3^H]NAMH) displacement assay on H_3_R-expressing HEK293T cell homogenates. The first step of a covalent ligand binding is the reversible binding to the binding site, which is followed by the formation of the covalent bond in case of appropriate orientation of the reactive warhead [13]. Consequently, binding equilibrium cannot be established and therefore an ‘apparent’ affinity (pK_i_) is reported for potential covalent ligands **1**, **20**–**23**, **43** and **44** (Table 1). To identify potential irreversible binders, 100 µM ligand was pre-incubated with human H_3_R (hH_3_R)-expressing cell homogenates for 1 h at 25 °C prior to vigorous washout of the compounds. The washed membranes were then subjected to a [^3^H]NAMH displacement assay to determine the amount of receptors that are still available for [^3^H]NAMH binding, which is taken as a measure of potential covalent H_3_R labeling (Table 1).

We expected that H_4_R covalent binder **1** [15] can also covalently bind H_3_R due to the high sequence similarity with H_4_R and the conservation of the anchor residue C^3.36^ [29]. Although ligands **1** and **2** showed both comparable micromolar affinity for hH_4_R (pK_i_ 6.3 and 6.4, respectively) [15], ligand **2** showed a remarkable increase in H_3_R binding affinity (pK_i_: 7.1), while **1** showed only weak H_3_R affinity (pK_i_ < 5). Despite its weak apparent affinity, binding of [^3^H]NAMH to H_3_R-expressing cell homogenates pre-incubated with **1** after washout resulted in only 41% occupancy of the receptor population by [^3^H]NAMH, suggesting that **1** irreversibly binds to hH_3_R since binding of its non-covalent analog **2** is reversible (Table 1).

In the first design cycle, two H_3_R aminopyrimidine anchor-moieties (R^1^ = B or C) were combined with an ethenyl-warhead at Position 2 of pyrimidine core (R^2^) and an *i*Pr moiety as R^3^ (Table 1). Good binding affinity was obtained for the amino-derivative **10** while substitution at the R^2^ position by an alkyl (**34**, **35**), as well as ethenyl moiety (**21**), significantly reduced the binding affinity (pK_i_ < 5). Washout of **10**, **34**, **35** and **21** all resulted in high occupancy of the H_3_R with [^3^H]NAMH, indicating that receptors are not covalently labeled by **21** nor, as expected, by its non-covalent analogs **10**, **34** and **35**. To improve H_3_R binding affinity, the same compound set (R^3^ = *i*Pr; R^2^ = NH_2_, Me, Et, ethenyl) was synthesized with a cyclobutyl-piperazine moiety as anchor (R^1^ = C). Although the affinity of the amino-analog **11** was reduced compared to **10**, the affinities of non-covalent alkyl derivatives **36** and **37** as well as of ethenyl-derivative **22** increased to the micromolar range with respect to their counterparts in series B (Table 1). Washout experiments on non-covalent analogs **11**, **36** and **37** show reversibility, while **22** decreased the amount of receptors available for [^3^H]NAMH binding, indicating that a covalent interaction had been formed between **22** and H_3_R. To further improve both affinity and covalent labeling, optimization of core substitution (R^3^) and warhead (R^2^) were undertaken in the second design cycle. Reducing the *i*Pr moiety to a smaller Me substituent (R^3^ = Me) resulted in similar binding affinities for both the non-covalent (compare **12** to **11**) as well as covalent ethenyl (compare **23** to **22**) analog. Similar to **22** and **11**, compound **23** led to covalent labeling whereas **12** did not. In contrast, the ethynyl analog **20** displayed poor affinity and is incapable of inhibiting [^3^H]NAMH binding after washout, suggesting lack of irreversible binding presumably due to inappropriate orientation of the reactive electrophilic carbon. We explored more reactive warheads in order to improve upon the covalent H_3_R labeling by **22** and **23**.

Synthesis of analogs of **22** and **23** with isothiocyanate- and chloromethylketone warheads was attempted, but combining these warheads with the electron-withdrawing pyrimidine core was synthetically challenging. Therefore, in the third and final design cycle, the pyrimidine core was changed to a phenyl group. While use of the phenyl core still resulted in micromolar affinities for non-covalent amino analog **42**, the apparent affinity of potential covalent derivatives **43** and **44** was improved with respect to **22** and **23** (Table 1). Non-covalent control **42** and chloroacetamide **43** showed similarly low labeling in washout experiments, for the latter suggesting inappropriate orientation of the electrophilic carbon or low reactivity. Gratifyingly, though, after washout of **44** only 15% of the receptor population was available for [^3^H]NAMH binding, suggesting that an efficient formation of a covalent interaction with the H_3_R was formed.

### 2.4. Covalent Binding to Glutathione and Nonapeptide

**Figure 3:**
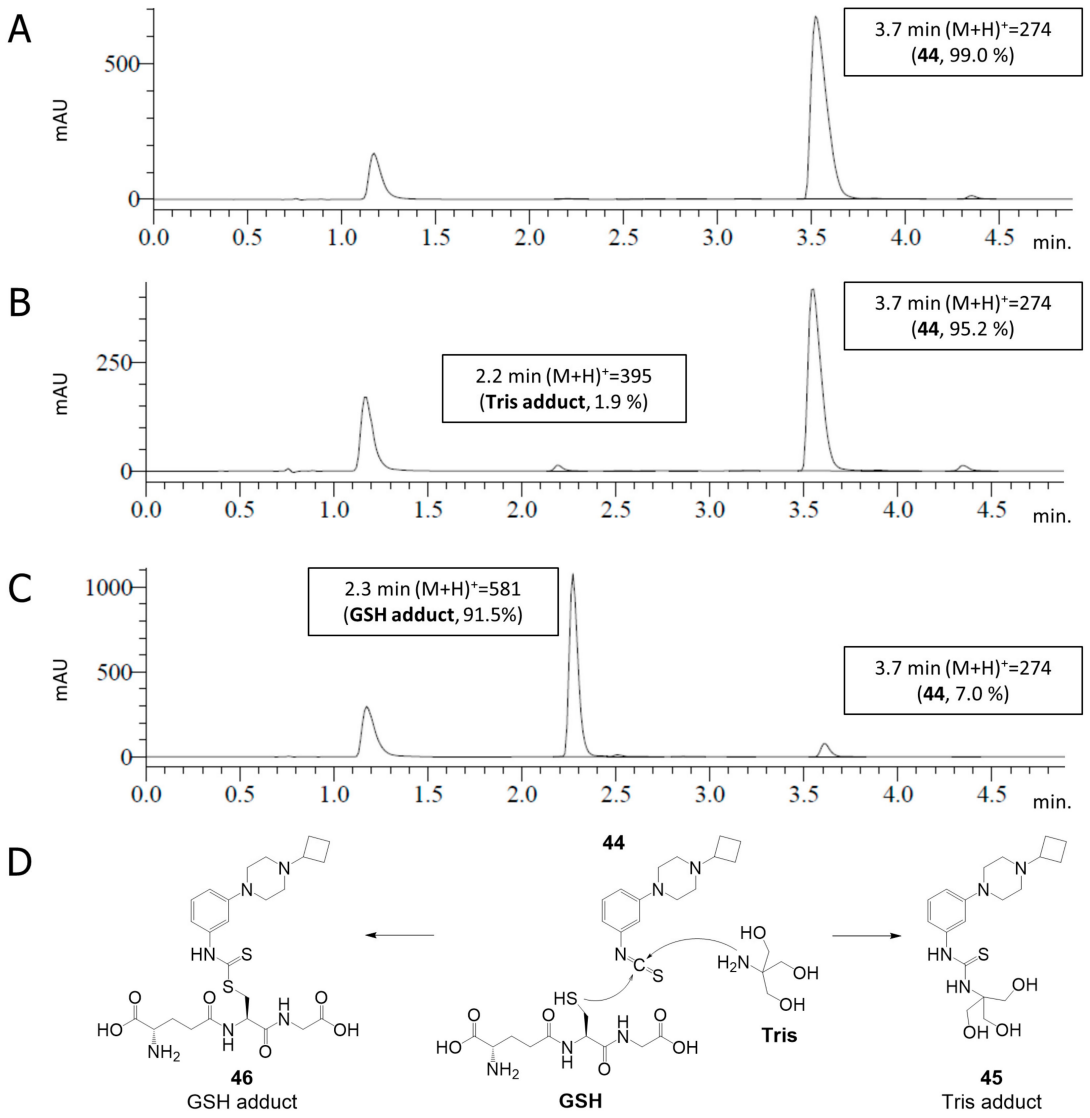
LC-MS analysis of **44** (0.40 mM) in 50 mM Tris-HCl pH 7.4 without (**A**,**B**) and with (**C**) GSH (1.2 mM). The mixtures were incubated for 10 min (**A**,**C**) or 100 min (**B**) at rt. The incubated mixtures were analysed by LC-MS. A signal from the DMSO used for the stock solution of **44** is visible at 1.2 min. (**D**) Postulated identified products.

Given the suggested covalent binding to the H_3_R (Table 1), key isothiocyanate **44** was tested for its chemical stability and reactivity with glutathione (GSH) under the conditions of the pharmacological experiments. Compound **44** (0.40 mM) was dissolved in Tris buffer (50 mM Tris-HCl, pH 7.4) in absence or presence of GSH (1.2 mM). The mixtures were incubated for 10–100 min at room temperature and analysed by LC-MS (Figure 3). Interestingly, the stability experiments showed a very slow reaction with Tris. That is, while **44** remained intact after 10 min incubation (Figure 3A), a very minor adduct of **44** and Tris (**45**) was visible (1.9%) after 100 min incubation (Figure 3B). Indeed, phenyl isothiocyanates are known to display reactivity towards amines and thiols albeit at different rates (vide supra) [12,32], but exert low reactivity towards alcohols [38]. Therefore, we postulate that the isothiocyanate moiety of **44** binds the amino group of Tris to afford adduct **45** (Figure 3D). Yet, the still high integrity of **44** (95%) at the pharmacologically relevant incubation time (100 min) indicates an appropriate stability of **44** in buffer for our pharmacology assays. Co-incubation with GSH in a 1:3 (**44**:GSH) molar ratio shows high reactivity, with the GSH adduct **46** visible at high conversion (91.5%) after 10 min incubation (Figure 3C). The reaction of **44** with GSH is postulated to proceed via the thiol group (Figure 3D).

**Figure 4:**
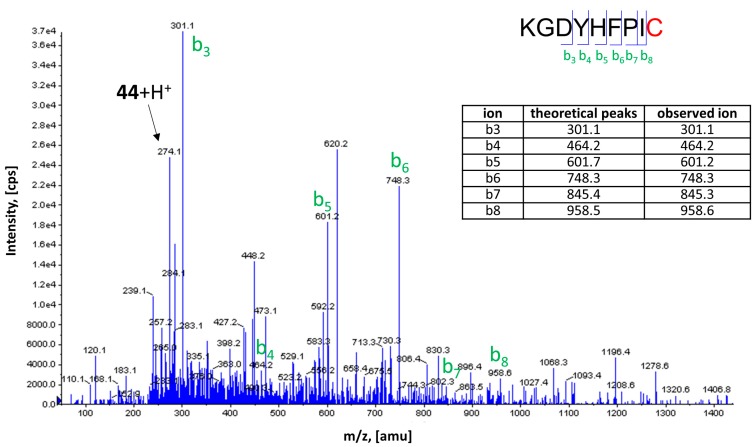
MS/MS spectrum of NP modified by **44** (*m*/*z* 676.5, see Figure S1A). Two-fold charged species were examined.

The selectivity of **44** toward nucleophilic residues was further investigated with an oligopeptide KGDYHFPIC. This nonapeptide (NP) is designed to harbor an array of nucleophilic residues, i.e., cysteine, lysine, tyrosine, aspartate and histidine [25]. Analysis by LC-MS/MS can identify the binding to a particular residue from fragmentation patterns. NP and **44** were incubated in phosphate-buffered saline pH 7.4 with 10% MeCN at rt for 16 h. LC-MS analysis indicates a reaction with the NP as evidenced by a NP+**44**+H+ ion at *m*/*z* 1351.5 as well as its doubly-charged ion at *m*/*z* 676.5 in the peak at 4.3 min (Figure S1A). The MS/MS spectrum of the two-fold charged NP modified by **44** (*m*/*z* 676.5) shows a fragmentation pattern indicative of the cysteine monoadduct of **44** (Figure 4), suggesting selective binding of the isothiocyanate moiety of **44** to the cysteine residue. The non-covalent analog **42** does not show any reaction with NP (Figure S1B).

### 2.5. Covalent Labeling of the H_3_R by **44**

**Figure 5:**
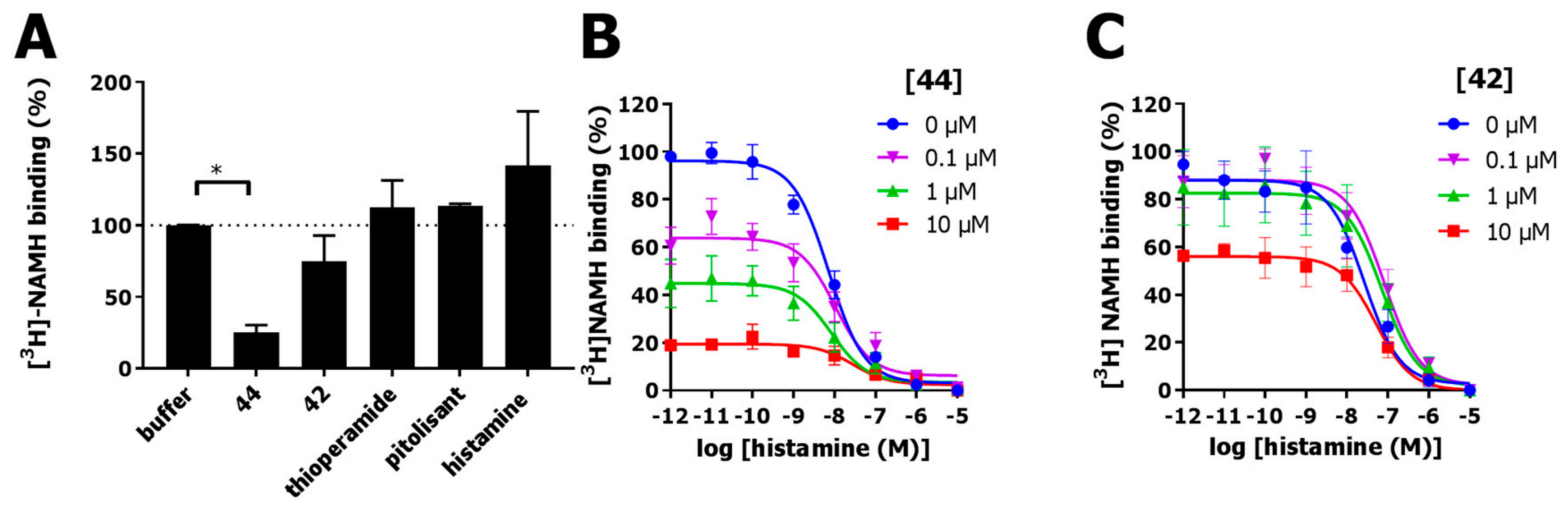
(**A**) Binding of [^3^H]NAMH to hH_3_R-expressing HEK293T cell homogenates that were pre-incubated with vehicle, 10 µM **44**, 10 µM **42** or 10 µM of non-covalent reference H_3_R ligands thioperamide, pitolisant and histamine for 1 h at 25 °C, followed by washout. (**B**,**C**) Radioligand displacement curves of [^3^H]NAMH with histamine on cell homogenates preincubated for 1 h at 25 °C with increasing concentrations of (**B**) **44** or (**C**) **42**. After thorough washing pretreated cell homogenates were subjected to displacement of [^3^H]NAMH with increasing histamine concentrations. Data are normalized to specific binding of vehicle treated cell homogenates. Data shown are pooled data mean ± S.E.M. of at least three experiments performed in triplicate. * *p* < 0.05 (one-way ANOVA with Fisher’s LSD test).

To further validate that **44** binds irreversibly to the H_3_R, recovery of [^3^H]NAMH binding after washout of H_3_R-expressing cell homogenates preincubated with 10 µM **44** or **42** was compared to washout of three reference non-covalent H_3_R ligands (namely thioperamide, pitolisant, and histamine) with relatively long to short residence times [39]. Indeed, 10 µM **44** could significantly (*p* < 0.05, one-way ANOVA with Fisher’s LSD test) inhibit recovery of [^3^H]NAMH binding after washout, while washout of H_3_R expressing cell homogenates preincubated with **42**, histamine, pitolisant, or thioperamide recovered [^3^H]NAMH occupancy to vehicle levels (Figure 5A).

Moreover, after washout of cell homogenates pretreated with 0.1–10 µM **44** a concentration-dependent decrease in [^3^H]NAMH occupancy of the H_3_R up to 20% is observed**,** while the affinity of histamine was unaffected (Figure 5B). In contrast, pretreatment with non-covalent analog **42** showed no significant decrease in [^3^H]NAMH binding up to 1 µM (Figure 5C). However, at 10 µM **42** only 56% [^3^H]NAMH labeling could be observed despite three vigorous wash steps to remove the ligand.

Due to high homology between H_3_R and H_4_R binding of **42** and **44** was evaluated in a NanoBRET-based fluorescent ligand displacement assay on the Nanoluc-H_4_R. Both **42** and **44** were incapable of displacing the fluorescent tracer clobenpropit-BODIPY from the Nanoluc-H_4_R up to a concentration of 10 µM (Figure S2), indicating that binding of **44** and **42** is selective to H_3_R.

### 2.6. Functional Characterization

**Figure 6:**
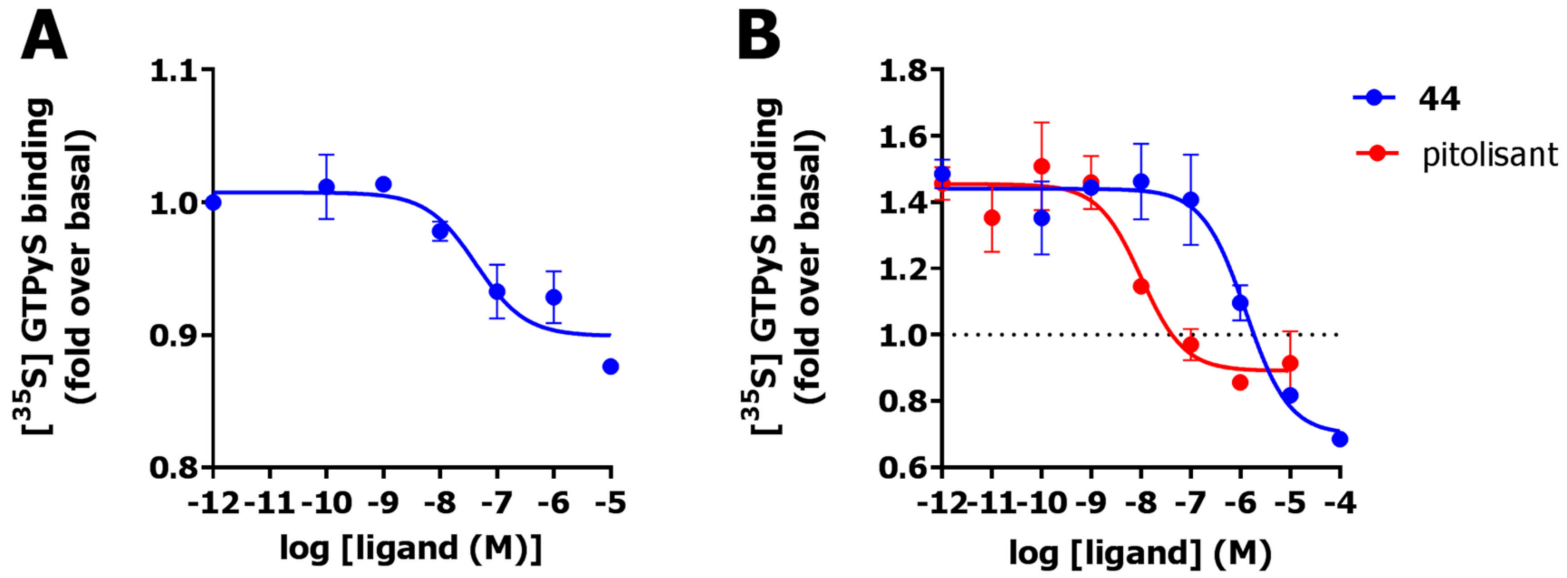
Functional characterization of **44** as measured by [^35^S]GTPγS binding to hH_3_R expressing HEK293T cell homogenates at 25 °C for 1 h. (**A**) Inhibition of basal hH_3_R activity by **44**. (**B**) Inhibition of 31.6 nM immepip-induced hH_3_R activity by **44** or pitolisant.

Functional characterization of **44** in a [^35^S]GTPyS assay showed that **44** inhibited basal H_3_R signaling with a pEC_50_ value of 7.1 ± 0.3 (Figure 6A). Moreover, **44** could inhibit H_3_R activity induced by immepip (31.6 nM) to levels below basal signaling with a pIC_50_ value of 6.1 ± 0.1, suggesting that it might act as inverse agonist (Figure 6B). In comparison, the marketed H_3_R inverse agonist pitolisant more potently inhibited immepip-induced H_3_R activity (pIC_50_ 8.1 ± 0.1), however, inhibition of basal H_3_R signaling was more pronounced for **44**.

## 3. Discussion

Covalent small-molecule ligands can be useful tools for the better understanding of GPCR structure and function [14,17]. Despite intense investigations on the histamine receptor GPCR family [2], histamine receptors have only been targeted with covalent ligands sporadically [15,17]. The aim of the current study was the identification of a covalent H_3_R ligand. The high homology of H_3_R and H_4_R [28] suggested that the published covalent partial H_4_R agonist VUF14480 [15] was a suitable starting point. The first design cycle resulted in a cyclobutyl-piperazine anchor moiety, which was combined with a change in warhead in the second design cycle, resulting in Michael acceptor vinyl-ligands **22** and **23** with micromolar apparent affinity for H_3_R. In a subsequent scaffold hopping, the pyrimidine core was changed to phenyl in the third design cycle, providing the phenyl-isothiocyanate **44** with subnanomolar apparent affinity for H_3_R and no measurable interaction with H_4_R up to least 10^−5^ M.

The alkyl-isothiocyanate moiety as reactive warhead has been regularly used for covalent targeting, e.g., for labeling of cannabinoid receptors [18,40,41], and were found to predominantly interact with cysteine residues. Under physiological conditions alkyl-isothiocyanate rapidly form adducts with cysteines, however, in the presence of lysines, a slow but irreversible reaction could also form lysine adducts [32] as was observed with the some reactive phenyl isothiocyanates [33]. According to our reactivity/stability assays, phenyl-isothiocyanate **44** forms an adduct with cysteine in a rapid reaction without significant aqueous decomposition. A more in-depth nonapeptide assay shows that the reaction is selective for cysteine in the presence of other nucleophilic residues, and formation of lysine adducts did not significantly occur during an incubation period of 16 h.

Determination of washout resistance by radioligand binding is a frequently used method for the validation of a postulated irreversible interaction between a potential covalent ligand and the targeted receptor [15,16,17,18,19,20,21,22,23]. In this experimental design, the bound ligand **44** was washed out after the preincubation with hH_3_R expressing cell homogenate and showed a concentration-dependent labeling of the receptor, while the non-covalent control **42** and reference ligands recovered radioligand binding to vehicle levels. This result indicates the irreversible nature of **44** binding to the binding pocket.

The unraveling of the crystal structure of H_1_R [42] has significantly impacted research on the histamine receptor family. However, the moderate similarity between H_1_R and H_3_R causes limitations in obtaining H_3_R models based on the structure of H_1_R [29]. Covalent ligands have been shown to facilitate GPCR crystallization [14,43]. We therefore believe that the identified covalent inverse agonist **44** could aid in stabilizing the H_3_R in the inactive conformation and as such function as a tool compound in H_3_R crystallization. The covalent binder **44** might also serve as a useful extension of the diverse H_3_R compound set for studying H_3_R signaling [39,44].

In conclusion, we designed and synthesized potential covalent ligands for H_3_R. Three successive design cycles led to identification of **44** (VUF15662) as an irreversible H_3_R binder. Tool compound **44** can be used to stabilize the inactive conformation of hH_3_R and to evaluate the effects of long-term blockage of H_3_R signaling.

## 4. Materials and Methods

### 4.1. Pharmacology

#### 4.1.1. Materials

[^3^H]NAMH (specific activity: 79.7 Ci/mmol) and [^35^S]GTPyS (specific activity 2200 Ci/mmol) were purchased from Perkin Elmer (Boston, MA, USA). All other chemicals were obtained from commercial suppliers and were of analytical grade.

#### 4.1.2. Cell Culture and Transfection

Human embryonic kidney 293T cells (HEK293T) (ATCC, Manassas, VA, USA) were cultured in DMEM (Gicbo, Thermo Fisher Scientific, Waltham, MA, USA) supplemented with 10% FBS (Bodinco, Alkmaar, the Netherlands) and 1% pen/step (Gibco, Thermo Fisher Scientific, Waltham, MA, USA). Two million cells per 10 cm^2^ dishes were plated 24 h prior to transfection. Cells were transfected using the polyethyleneimine (PEI) method [44] with 2500 ng cDNA encoding the hH_3_R and 2500 ng empty plasmid pcDEF3.

#### 4.1.3. Preparation of Cell Homogenates

Cell homogenates expressing the hH_3_R were harvested 48 h after transfection as reported previously [39].

#### 4.1.4. Radioligand Displacement Assays

[^3^H]NAMH displacement assays were perform in binding buffer [50 mM Tris-HCl pH 7.4, 25 °C] by co-incubation of ~2 nM [^3^H]NAMH, increasing concentration of unlabeled ligand and cell homogenates expressing the hH_3_R. Assay mixture was incubated for 2 h at 25 °C before rapid filtration over a PEI-coated GF/C filter with a Perkin Elmer filtermate harvester. Filterplate was dried and 300 min after 25 µL Microsint O was added filterbound radioactivity was measured with a Microbeta scintillation counter (Perkin Elmer).

#### 4.1.5. Receptor Recovery Assay

To assess covalent binding of unlabeled ligands, hH_3_R expressing cell homogenates were pre-incubated for 1 h with either Tris buffer [50 mM Tris-HCl pH 7.4], 10 µM, 1 µM or 0.1 µM unlabeled ligand, or 100 µM unlabeled ligand for screening, in a thermoshaker (1000 RPM, 25 °C). Subsequently, cell homogenates were vigorously washed according to the procedure described in Nijmeijer et al. (2013) [15] and pelleted cell homogenates were reconstituted in Tris buffer and subjected to radioligand displacement assay (vida supra).

#### 4.1.6. [^35^S]GTPγs Assay

In [^35^S]GTPyS assay, hH_3_R expressing cell homogenates (20 µg/well) were incubated in GTPγs buffer [50 mM HEPES, 150 mM NaCl, 10 mM MgCl_2_, 4 µM GDP, 0.2 µg saponin, pH 7.4] with ~0.5 nM [^35^S]GTPγS and increasing ligand concentrations (10^−4^ M to 10^−12^ M) for 1 h at 25 °C. Reaction was stopped by rapid filtration of a GF/B filter with a Perkin Elmer filtermate harvester. Filterplate was dried and 25 µL Microsint O was added to measure filterbound radioactivity with a Microbeta scintillation counter (Perkin Elmer) at 500 min.

#### 4.1.7. Chemical Stability and Reactivity of **44**

The reactivity of **44** (400 µM) was tested in the mixture of binding buffer (96% 50 mM Tris-HCl pH 7.4 and 4% DMSO) with or without GSH (1.2 mM) at rt. At various timepoints, the mixtures were directly injected and analysed by LC-MS, using the acidic mode elution programme described in the General information of the chemistry experimental section.

#### 4.1.8. Nonapeptide Assay

Analysis of NP (KGDYHFPIC) incubated (16 h, rt) with **44** or **42** was performed according to Ábrányi-Balogh et al. [25].

#### 4.1.9. Data Analysis

All data was analysed and statistical analysis performed using Graphpad prism 7.02 (Graphpad software inc, San Diego, USA). Competition binding curves were fitted using a one-site binding model and affinity (pK_i_) values were calculated using Cheng–Prusoff equation [45].

### 4.2. Modelling

The homology model of H_3_R was constructed according to Hauwert et al. [46].

The covalent compound docking (Figure 2) was performed using GOLD version 5.2.1 using the Piecewise Linear Potential (PLP) fitness function. A flood fill of the orthosteric binding site was used as reference for the docking site. The sulfur atom of C118^3.36^ was assigned as covalent anchor point and the pocket residues W110^3.28^, D114^3.32^, Y115^3.33^, E206^5.46^, W371^6.48^, and W402^7.43^ were treated as semi-flexible by sampling different rotamers from the GOLD rotamer library.

### 4.3. Chemistry

#### 4.3.1. General Information

Chemicals and solvents were obtained from commercial suppliers and were used without further purification. Dry DCM and dioxane were obtained from PureSolv solvent purification system by Inert^®^. All reactions were carried out under an inert N_2_ atmosphere. Microwave reactions were performed with Biotage Initiator microwave system. TLC analyses were performed with Merck F254 alumina silica plates using UV visualization or staining. Column purifications were carried out automatically using Biotage Isolera and Silicycle Ultra Pure silica gel. Melting point (Mp) for final compounds was determined using a Büchi M-565 melting point apparatus with a rate of 1 °C/min. NMR spectra were recorded on a Bruker 250 or 500 MHz spectrometer. Chemical shifts are reported in ppm (δ), and the residual solvent was used as internal standard (δ ^1^H NMR: CDCl_3_ 7.26; D_2_O 4.79; ^13^C NMR: CDCl_3_ 77.16). Data are reported as follows: chemical shift, multiplicity (s = singlet, d = doublet, t = triplet, q = quartet, p = pentet, hept = heptet, br = broad signal, m = multiplet, app = apparent), coupling constants (Hz) and integration. HRMS spectra were recorded on Bruker microTOF mass spectrometer using ESI in positive ion mode. Analytical HPLC-MS analyses were conducted using a Shimadzu LC-20AD liquid chromatograph pump system connected to a Shimadzu SPDM20A diode array detector with MS detection using a Shimadzu HPLC-MS 2010EV mass spectrometer. The column used is an Xbridge C18 5 mm column (50mm × 4.6 mm). *Acidic mode:* Solvent B (MeCN/0.1% formic acid) and solvent A (water/0.1% formic acid), flow rate of 1.0 mL/min with a run time of 8 min. For compounds which retention time (t_R_) was less than 1.5 min with acidic solvent system, a basic solvent system was used. *Basic mode:* Solvent B (MeCN/10% buffer), Solvent A (water/10% buffer). The buffer is a 0.4% (*w*/*v*) NH_4_HCO_3_ solution in water, adjusted to pH 8.0 with NH_4_OH. The analysis was conducted using a flow rate of 1.0 mL/min with a total run time of 8 min. *Gradient settings* (basic and acidic system): start 5% B, linear gradient to 90% B in 4.5 min, then isocratic for 1.5 min at 90% B, then linear gradient to 5% B in 0.5 min, then isocratic for 1.5 min at 5% B. Unless specified otherwise, all compounds have a purity of ≥95%, calculated as the percentage peak area of the analysed compound by UV detection at 254 nm. Yields reported are not optimized. The compounds described in Table 1 were checked for the presence of PAINS substructures as described by Baell and Holloway [47], and no PAINS substructures were identified.

#### 4.3.2. Synthesis

The syntheses of final compounds **10**–**12**, **20**–**23**, **34**–**37** and **43** are described in the Supplementary Materials.

*Tert-butyl 4-(3-nitrophenyl)piperazine-1-carboxylate* (**39**). A solution of bromide **38** (808 mg, 4.00 mmol) and NaO*t*Bu (461 mg, 4.80 mmol) in dioxane (15 mL) was degassed with N_2_ for 10 min. Next, Pd_2_(dba)_3_ (183 mg, 0.20 mmol), Xantphos (347 mg, 0.60 mmol) and *tert*-butyl piperazine-1-carboxylate (1.12 g, 6.00 mmol) were added. The reaction mixture was heated for 1 h at 80 °C under microwave irradiation. The reaction mixture was diluted with water (40 mL) and extracted with DCM (3 × 30 mL). The combined organic layers were dried over Na_2_SO_4_, filtered and concentrated in vacuo. Purification by flash chromatography (heptane:EtOAc 5:0 to 4:1) gave the title compound as an orange solid (700 mg, 57%). ^1^H NMR (500 MHz, CDCl_3_) δ 7.72 (t, *J.* = 2.2 Hz, 1H), 7.71–7.67 (m, 1H), 7.39 (t, *J.* = 8.2 Hz, 1H), 7.20 (dd, *J.* = 8.3, 2.1 Hz, 1H), 3.61 (t, *J.* = 5.2 Hz, 4H), 3.24 (t, *J.* = 5.1 Hz, 4H), 1.49 (s, 9H). HPLC-MS (acidic mode): t_R_ = 5.3 min, purity: 98.6%, [M + H]^+^: 308.

*1-(3-Nitrophenyl)piperazine* (**40**). To a solution of carbamate **39** (1.20 g, 3.90 mmol) in dioxane (20 mL) was added HCl in dioxane (4N, 9.75 mL, 39.0 mmol). The reaction mixture was stirred for 1 h at rt. The solvent was removed under reduced pressure. The residue was mixed with satd. aq. Na_2_CO_3_ (40 mL) and extracted with DCM (3 × 30 mL). The combined organic layers were dried over Na_2_SO_4_, filtered and concentrated in vacuo. The title compound was obtained as a brown solid (633 mg, 78%). ^1^H NMR (500 MHz, CDCl_3_) δ 7.71 (s, 1H), 7.66 (d, *J.* = 8.0 Hz, 1H), 7.37 (t, *J.* = 8.2 Hz, 1H), 7.19 (d, *J.* = 8.3 Hz, 1H), 3.28–3.21 (m, 4H), 3.10–3.02 (m, 4H). HPLC-MS (acidic mode): t_R_ = 2.6 min, purity: 97.3%, [M + H]^+^: 208.

*1-Cyclobutyl-4-(3-nitrophenyl)piperazine* (**41**). To a solution of amine **40** (630 mg, 3.04 mmol) in DCM (20 mL) was added cyclobutanone (273 μL, 3.66 mmol). After 10 min of stirring at rt, NaBH(OAc)_3_ (966 mg, 4.56 mmol) was added and the resulting mixture was stirred at rt overnight. The reaction mixture was quenched with satd. aq. Na_2_CO_3_ (30 mL) and extracted with DCM (3 × 15 mL). The combined organic phases were dried over Na_2_SO_4_, filtered and concentrated in vacuo. Purification by flash chromatography (DCM:MeOH 20:0 to 19:1) gave the title compound as a yellow oil (600 mg, 76%). ^1^H NMR (500 MHz, CDCl_3_) δ 7.71 (t, *J.* = 2.3 Hz, 1H), 7.65 (dd, *J.* = 8.1, 1.5 Hz, 1H), 7.37 (t, *J.* = 8.2 Hz, 1H), 7.18 (dd, *J.* = 8.3, 2.1 Hz, 1H), 3.38–3.23 (m, 4H), 2.80 (p, *J.* = 7.9 Hz, 1H), 2.51 (t, *J.* = 5.1 Hz, 4H), 2.13–2.04 (m, 2H), 2.00–1.87 (m, 2H), 1.81–1.57 (m, 2H, overlaps with residual water). HPLC-MS (acidic mode): t_R_ = 2.8 min, purity: 97.8%, [M + H]^+^: 262.

*3-(4-Cyclobutylpiperazin-1-yl)aniline* (**42**). To a solution of nitrocompound **41** (59 mg, 0.23 mmol) in MeOH (2 mL) was added HCOONH_4_ (71 mg, 1.13 mmol) as a solid followed by a suspension of Pd/C (10%, 24 mg) in water (2 mL). The reaction mixture was stirred at rt overnight. The mixture was filtered over Celite and the filtrate was concentrated in vacuo. The residue was diluted with aq. Na_2_CO_3_ (1.0 M, 10 mL) and extracted with DCM (3 × 5 mL). The combined organic layers were dried over Na_2_SO_4_, filtered and concentrated in vacuo. The crude product was purified by flash chromatography (DCM:MeOH 20:0 to 19:1). The selected fractions were collected and the solvents were evaporated. The residue was dissolved in aq. HCl (1.0 M, 10 mL), washed with EtOAc (2 × 5 mL) and *c*-hexane (3 × 10 mL). The pH of the aqueous layer was adjusted to 10 with satd. aq. Na_2_CO_3_ and extracted with EtOAc (3 × 10 mL). The combined organic phases were dried over Na_2_SO_4_, filtered and concentrated in vacuo. The title compound was obtained as an off-white solid (13 mg, 25%). Mp: 92.5–92.7 °C. ^1^H NMR (500 MHz, CDCl_3_) δ 7.04 (t, *J.* = 8.0 Hz, 1H), 6.36 (dd, *J.* = 8.2, 2.3 Hz, 1H), 6.25 (t, *J.* = 2.3 Hz, 1H), 6.21 (dd, *J.* = 7.7, 2.0 Hz, 1H), 3.59 (br, 2H), 3.26–3.10 (m, 4H), 2.78 (p, *J.* = 7.9 Hz, 1H), 2.56–2.37 (m, 4H), 2.12–2.02 (m, 2H), 2.00–1.88 (m, 2H), 1.80–1.64 (m, 2H). ^13^C NMR (126 MHz, CDCl_3_) δ 152.6, 147.4, 130.0, 107.1, 107.1, 103.0, 60.4, 49.6, 48.8, 27.1, 14.4. HPLC-MS (basic mode): t_R_ = 4.1 min, purity: 98.4%, [M + H]^+^: 232. HR-MS [M + H]^+^ calcd for C_14_H_22_N_3_^+^: 232.1808, found 232.1818.

*1-Cyclobutyl-4-(3-isothiocyanatophenyl)piperazine* (**44**). To an ice-cold mixture of aniline **42** (46 mg, 0.20 mmol) in DCM (2 mL) and aq. NaHCO_3_ (1.0 M, 30 mL) was added dropwise a solution of CSCl_2_ (18 μL, 0.24 mmol) in DCM (1 mL). The reaction mixture was stirred for 1 h at rt. The reaction mixture was diluted with water (10 mL) and extracted with DCM (3 × 5 mL). The combined organic layers were dried over Na_2_SO_4_, filtered and concentrated in vacuo. Purification by flash chromatography (DCM:MeOH 20:0 to 19:1) gave the title compound as a white solid (35 mg, 64%). The compound is stable as a solid in the freezer (−20 °C) over a period of at least 2 years as judged by NMR and LC-MS analysis. Mp: 79.7–80.1°C. ^1^H NMR (500 MHz, CDCl_3_) δ 7.18 (t, *J.* = 8.1 Hz, 1H), 6.81 (dd, *J.* = 8.5, 2.4 Hz, 1H), 6.72–6.65 (m, 2H), 3.23–3.15 (m, 4H), 2.77 (p, *J.* = 7.9 Hz, 1H), 2.50–2.42 (m, 4H), 2.11–2.02 (m, 2H), 1.96–1.86 (m, 2H), 1.79–1.64 (m, 2H). ^13^C NMR (126 MHz, CDCl_3_) δ 152.2, 134.2, 131.9, 130.0, 116.5, 114.9, 112.6, 60.3, 49.3, 48.3, 27.1, 14.4. HPLC-MS (acidic mode): t_R_ = 3.6 min, purity: 98.9%, [M + H]^+^: 274. HR-MS [M + H]^+^ calcd for C_15_H_20_N_3_S^+^: 274.1372, found 274.1374.

## Figures and Tables

**Table 1:** (Apparent) binding affinity (pK_i_) of ligands for hH_3_R expressed on HEK293T cell homogenates, and the amount of unlabeled receptors remaining after 1 h preincubation of hH_3_R expressing membranes with 100 µM ligand and subsequent washout as determined by radioligand binding. Ligands are considered potential covalent binders when % unlabeled receptors remaining after 1 h pre-incubation with ligand was <50%. Data are mean ± S.E.M of 3 experiments performed in triplicate with the exception of **1** for which mean ± SD is reported for two experiments performed in triplicate.

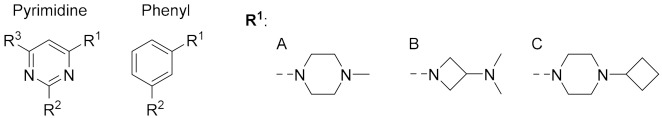						
Cmpd.	Central Ring	R^1^	R^2^	R^3^	pK_i_	% Unlabeled Receptors
**2**	Pyrimidine	A	Me	phenyl	7.1 ± 0.1	84 ± 16
**1**	Pyrimidine	A	CH=CH_2_	phenyl	< 5	41 ± 6 (SD)
**10**	Pyrimidine	B	NH_2_	*i*Pr	7.3 ± 0.1	100 ± 0.0
**34**	Pyrimidine	B	Me	*i*Pr	< 5	87 ± 13
**35**	Pyrimidine	B	Et	*i*Pr	< 5	93 ± 7
**21**	Pyrimidine	B	CH=CH_2_	*i*Pr	< 5	85 ± 15
**11**	Pyrimidine	C	NH_2_	*i*Pr	6.7 ± 0.1	96 ± 4
**36**	Pyrimidine	C	Me	*i*Pr	5.9 ± 0.1	96 ± 3
**37**	Pyrimidine	C	Et	*i*Pr	5.5 ± 0.1	89 ± 11
**22**	Pyrimidine	C	CH=CH_2_	*i*Pr	5.5 ± 0.0	41 ± 9
**12**	Pyrimidine	C	NH_2_	Me	6.5 ± 0.1	91 ± 5
**23**	Pyrimidine	C	CH=CH_2_	Me	5.4 ± 0.1	40 ± 8
**20**	Pyrimidine	C	C≡CH	Me	< 5	80 ± 2
**42**	Phenyl	C	NH_2_	-	5.9 ± 0.3	72 ± 8
**43**	Phenyl	C	NH-CO-CH_2_-Cl	-	6.2 ± 0.1	86 ± 5
**44**	Phenyl	C	N=C=S	-	6.5 ± 0.3	15 ± 2

## Supplementary Materials

The following are available online. LC-MS analysis of nonapeptide assays with **44** and **42**; Nluc-H_4_R displacement of clobenpropit-BODIPY by **42**, **44** and reference ligands; syntheses of final compounds **10**–**12**, **20**–**23**, **34**–**37** and **43**; LC-MS chromatogram and ^1^H, ^13^C, HSQC and HMBC NMR spectra for **44** and **42**.

## Abbreviations

[^3^H]NAMH	[^3^H]N-α-methylhistamine
ANOVA	analysis of variance
DCM	Dichloromethane
DIPEA	*N,N*-Diisopropylethylamine
DME	1,2-Dimethoxyethane
DMSO	dimethyl sulfoxide
GPCR	G protein-coupled receptor
GSH	glutathion
GTPγS	guanosine 5′-O-[gamma-thio]triphosphate
LSD	least significant difference
MeCN	Acetonitrile
MIDA	N-methyliminodiacetic acid
Mp	Melting point
NP	nonapeptide (KGDYHFPIC)
SAR	structure-activity relationship
satd. aq.	saturated aqueous
SD	standard deviation
SEM	standard error of mean
rt	room temperature
TCI	targeted covalent inhibitors
TEA	Triethylamine
Tris	2-Amino-2-(hydroxymethyl)propane-1,3-diol
μW	microwave reaction
wt	wild type
