# Supplementary material for: Covalent Inhibition of the Histamine H3 Receptor
Source: Molecules. 2019 Dec 11;24(24):4541. doi: 10.3390/molecules24244541 (PMC6943558; doi:10.3390/molecules24244541)
Supplement: Supplementary file 1 [file molecules-24-04541-s001.pdf]

Supplementary Materials for

Covalent inhibition of the histamine H_3 receptor

Gábor Wágner [1,†], Tamara A. M. Mocking [1,†], Albert J. Kooistra [1], Inna Slynko [1], Péter Ábrányi-Balogh [2], György M. Keserű [2], Maikel Wijtmans [1], Henry F. Vischer [1], Iwan J. P. de Esch [1] and Rob Leurs [1,*]

[1] Amsterdam Institute for Molecules, Medicines and Systems (AIMMS), Division of Medicinal Chemistry, Faculty of Science, Vrije Universiteit Amsterdam, De Boelelaan 1108, 1081 HZ Amsterdam, The Netherlands
[2] Medicinal Chemistry Research Group, Research Centre for Natural Sciences, Hungarian Academy of Sciences, Magyar tudósok krt 2, H-1117 Budapest, Hungary
* Correspondence: r.leurs@vu.nl; Tel.: +31-205987579
† These authors contributed equally to this work.

Contents

Page 3	**Figure S1:** LC-MS analysis of nonapeptide (NP) incubated with **44** and **42**.
Page 4	**Figure S2:** Displacement of clobenpropit-BODIPY by **42**, **44** and reference ligands clobenpropit and thioperamide from the Nluc-H_4R.
Page 5	**Synthesis of compounds 10-12, 20-23, 34-37 and 43**
Page 13	**Figure S3:** HPLC-MS chromatogram of compound **44**.
Page 14	**Figure S4:** ^{1}H NMR spectrum of compound **44** in $CDCl_3$.
Page 15	**Figure S5:** ^{13}C NMR spectrum of compound **44** in $CDCl_3$.
Page 16	**Figure S6:** HSQC NMR spectrum of compound **44** in $CDCl_3$.
Page 17	**Figure S7:** HMBC NMR spectrum of compound **44** in $CDCl_3$.
Page 18	**Figure S8:** HPLC-MS chromatogram of compound **42**.
Page 19	**Figure S9:** ^{1}H NMR spectrum of compound **42** in $CDCl_3$.
Page 20	**Figure S10:** ^{13}C NMR spectrum of compound **42** in $CDCl_3$.
Page 21	**Figure S11:** HSQC NMR spectrum of compound **42** in $CDCl_3$.
Page 22	**Figure S12:** HMBC NMR spectrum of compound **42** in $CDCl_3$.

Nonapeptide binding

A

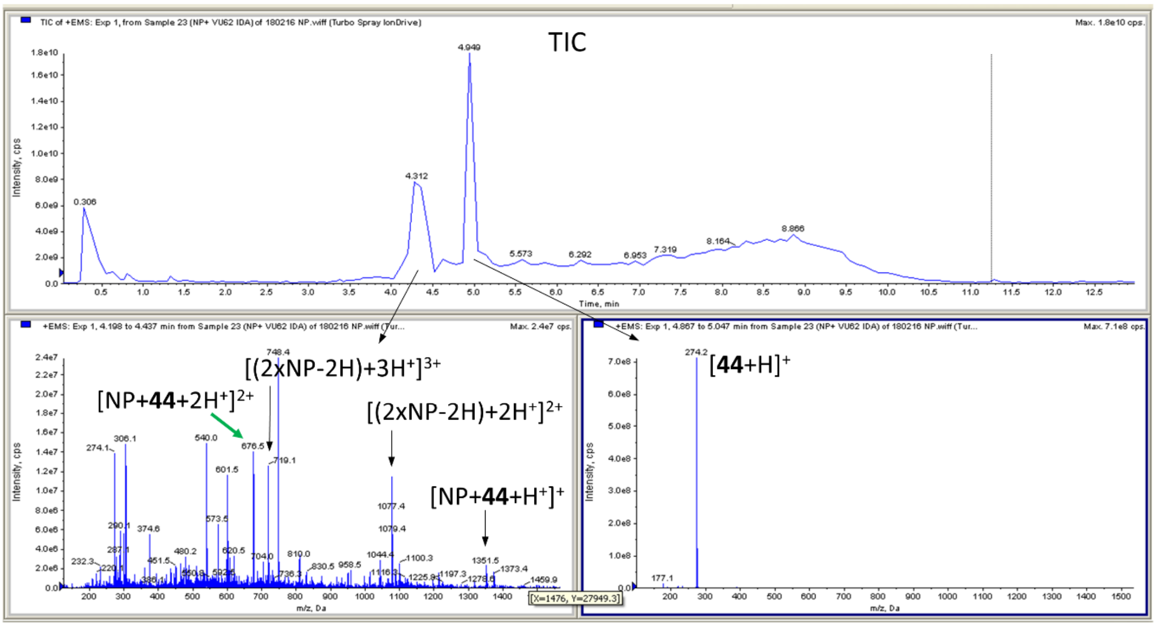

B

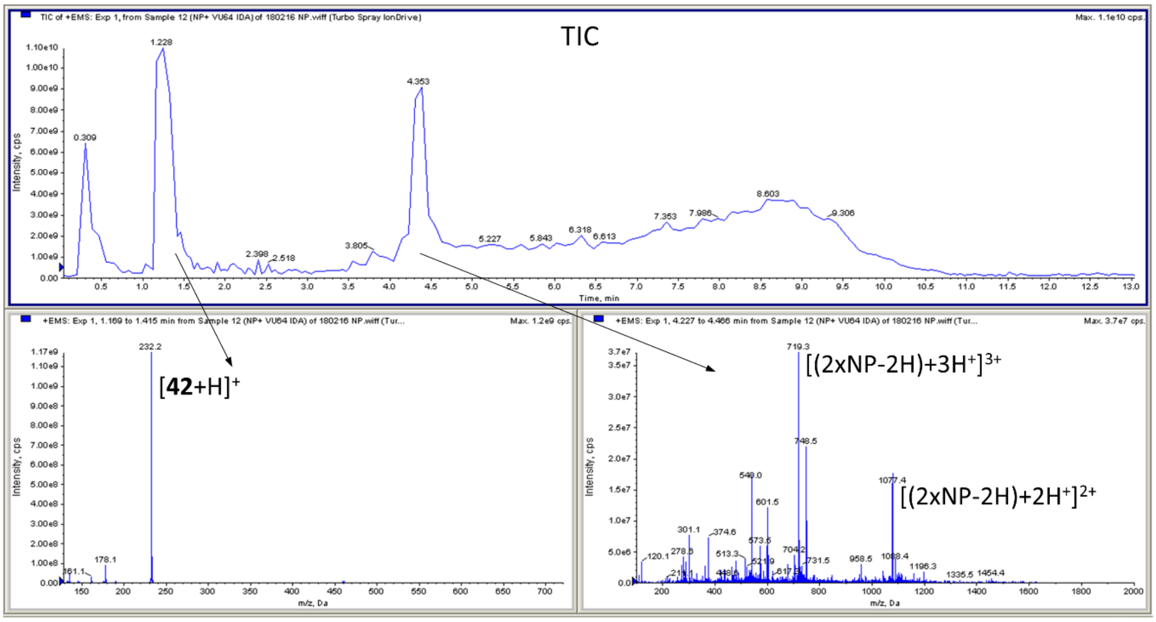

Figure S1. LC-MS analysis of nonapeptide (NP) incubated with (**A**) **44** and (**B**) **42** in PBS buffer pH 7.4 with 10 % MeCN at rt for 16 h. The formation of a NP disulfide is a competing background reaction. The green signal at m/z=676.5 in the lower left panel of Figure S1A was chosen for the MS/MS analysis shown in Figure 4 of the main text.

NanoBRET displacement assay

NanoBRET displacement assays were performed as previously described [1]. In brief, Nluc-H4R expressing HEK293T cells were incubated with 50 nM clobenpropit-BODIPY (HelloBio, UK) and increasing concentration of unlabeled ligand (10^{-5} M to 10^{-11} M) in HBSS for 2 h at 25°C. Subsequently, binding of clobenpropit-BODIPY was detected by addition of Nanoglo (Promega, UK) as BRET ratio between Nluc (460 ± 80 nm) and clobenpropit-BODIPY (>610 nm) using a PHERAstar plate reader (BMG, Germany).

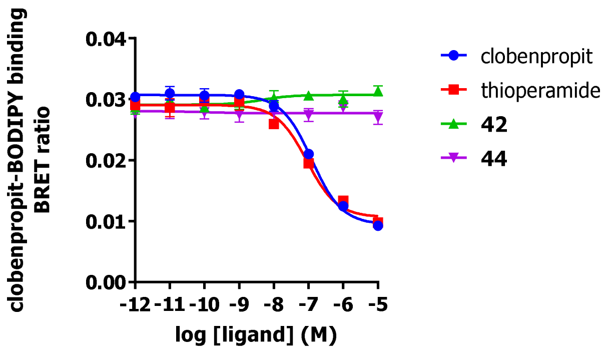

Figure S2. Displacement of clobenpropit-BODIPY by **42**, **44** and reference ligands clobenpropit and thioperamide from the Nluc-H4R.

Synthesis of final compounds 10-12, 20-23, 34-37 and **43**

4-Chloro-6-isopropylpyrimidin-2-amine (5). A preparation for this compound was previously published by us [46]. 2-Amino-6-isopropylpyrimidin-4(3*H*)-one **3** (4.87 g, 31.8 mmol) was dissolved in $POCl_3$ (40 mL, 0.43 mol). The mixture was heated to reflux for 3 h. The solvent was removed under reduced pressure. Ice (150 g) was carefully added to the residue. The pH of the mixture was adjusted to 9-10 with aq. NaOH (2.5 M). The mixture was extracted with DCM (3 x 100 mL). The combined organic phases were dried over Na_2SO_4, filtered and concentrated *in vacuo*. Purification by flash chromatography (DCM:MeOH 10:0 to 9:1) gave the title compound as an off-white solid (1.41 g, 26 %). ^1H NMR (500 MHz, $CDCl_3$) δ 6.53 (s, 1H), 5.33 (br, 2H), 2.77 (hept, *J* = 6.9 Hz, 1H), 1.22 (d, *J* = 6.9 Hz, 6H). HPLC-MS (acidic mode): t_R = 3.5 min, purity: 96.8 %, $[M + H]^+$: 172.

4-Chloro-6-methylpyrimidin-2-amine (6). 2-Amino-6-methypyrimidin-4(3*H*)-one **4** (5.00 g, 40.0 mmol) was dissolved in $POCl_3$ (60 mL, 0.64 mol). The mixture was heated to reflux for 3 h. The solvent was removed under reduced pressure. Ice (150 g) was carefully added to the residue. The pH of the mixture was adjusted to 9-10 with aq. NaOH (2.5 M). The mixture was extracted with DCM (3 x 100 mL). The combined organic phases were dried over Na_2SO_4, filtered and concentrated *in vacuo*. The residue was recrystallized from EtOH. The title compound was obtained as off-white crystals (1.91 g, 33 %). ^1H NMR (250 MHz, $CDCl_3$) δ 6.54 (s, 1H), 5.32 (br, 2H), 2.32 (s, 3H). HPLC (acidic mode): t_R = 2.5 min, purity: >99 %, mass too low to be detectable under used analytical conditions.

***Tert*-butyl (1-(2-amino-6-isopropylpyrimidin-4-yl)azetidin-3-yl)(methyl)carbamate (7).** A preparation for this compound was previously published by us [46] .A microwave vial charged with chloride **5** (300 mg, 1.75 mmol), *tert*-butyl azetidin-3-yl(methyl)carbamate hydrochloride (389 mg, 1.75 mmol), DIPEA (0.61 mL, 3.50 mmol) and dioxane (10 mL) was heated for 30 min at 150 °C under microwave irradiation. The reaction mixture was diluted with water (20 mL) and extracted with DCM (3 x 15 mL). The combined organic layers were dried over Na_2SO_4, filtered and concentrated *in vacuo*. Purification by flash chromatography (DCM:MeOH 10:0 to 9:1) gave the title compound as a yellow oil (280 mg, 50 %). ^1H NMR (250 MHz, $CDCl_3$) δ 5.50 (s, 1H), 5.03 (br, 1H), 4.79 (br, 2H), 4.20 (t, *J* = 8.6 Hz, 2H), 4.07 – 3.95 (m, 2H), 2.93 (s, 3H), 2.65 (hept, *J* = 6.9 Hz, 1H), 1.46 (s, 9H), 1.20 (d, *J* = 6.9 Hz, 6H). HPLC-MS (acidic mode): t_R = 3.4 min, purity: >99 %, $[M + H]^+$: 322.

***Tert*-butyl 4-(2-amino-6-isopropylpyrimidin-4-yl)piperazine-1-carboxylate (8).** A microwave vial charged with chloride **5** (560 mg, 3.26 mmol), *tert*-butyl piperazine-1-carboxylate (608 mg, 3.26 mmol), DIPEA (0.57 mL, 3.26 mmol) and dioxane (15 mL) was heated for 30 min at 150 °C under microwave irradiation. The reaction mixture was diluted with water (30 mL) and extracted with DCM (3 x 20 mL). The combined organic layers were dried over Na_2SO_4, filtered and concentrated *in vacuo*. Purification by flash chromatography (DCM:MeOH 10:0 to 9:1) gave the title compound as an off-white solid (1.05 g) which has an LC purity of >99 % but an estimated purity > 90 % by ^1H NMR analysis. ^1H NMR (250 MHz, $CDCl_3$) δ 5.80 (s, 1H), 4.77 (br, 2H), 3.62 – 3.53 (m, 4H), 3.52 – 3.44 (m, 4H), 2.67 (hept, *J* = 6.9 Hz, 1H), 1.47 (s, 9H), 1.21 (d, *J* = 6.9 Hz, 6H). HPLC-MS (acidic mode): t_R = 3.8 min, purity: >99 %, $[M + H]^+$: 322.

***Tert*-butyl 4-(2-amino-6-methylpyrimidin-4-yl)piperazine-1-carboxylate (9).** A microwave vial charged with chloride **6** (1.15 g, 8.00 mmol), *tert*-butyl piperazine-1-carboxylate (1.49 g, 8.00 mmol), DIPEA (1.40 mL, 8.00 mmol) and dioxane (30 mL) was heated for 30 min at 150 °C under microwave irradiation. The reaction mixture was diluted with water (60 mL) and extracted with DCM (3 x 30 mL). The combined organic layers were dried over Na_2SO_4, filtered and concentrated *in vacuo*. Purification by flash chromatography (DCM:MeOH 20:0 to 19:1) gave the title compound as an off-white solid (2.01 g, 85 %). ^1H

NMR (250 MHz, $CDCl_3$) δ 5.82 (s, 1H), 4.76 (br, 2H), 3.62 – 3.52 (m, 4H), 3.52 – 3.40 (m, 4H), 2.22 (d, *J* = 0.5 Hz, 3H), 1.47 (s, 9H). HPLC-MS (acidic mode): t_R = 3.0 min, purity: >99 %, $[M + H]^+$: 294.

4-(3-(Dimethylamino)azetidin-1-yl)-6-isopropylpyrimidin-2-amine (10). A preparation for this compound was previously published by us [46]. A microwave vial charged with chloride **5** (248 mg, 1.44 mmol), N,N-dimethylazetidin-3-amine dihydrochloride (250 mg, 1.44 mmol), DIPEA (0.76 mL, 4.33 mmol) and dioxane (10 mL) was heated for 30 min at 150 °C under microwave irradiation. The reaction mixture was diluted with water (20 mL) and extracted with DCM (3 x 20 mL). The combined organic layers were dried over Na_2SO_4, filtered and concentrated *in vacuo*. Purification by flash chromatography (DCM:MeOH:TEA 100:0:0 to 90:9:1) gave the title compound as a white solid (220 mg, 65 %). Mp: 123.6-123.8 °C. ^1H NMR (500 MHz, $CDCl_3$) δ 5.49 (s, 1H), 4.87 (br, 2H), 4.04 (t, *J* = 7.8 Hz, 2H), 3.94 – 3.81 (m, 2H), 3.21 (p, *J* = 5.6 Hz, 1H), 2.65 (hept, *J* = 6.8 Hz, 1H), 2.20 (s, 6H), 1.20 (d, *J* = 6.8 Hz, 6H). ^{13}C NMR (126 MHz, $CDCl_3$) δ 174.0, 164.6, 162.1, 89.1, 56.2, 53.9, 41.8, 35.5, 21.7. HPLC-MS (basic mode): t_R = 3.4 min, purity: 97.8 %, $[M + H]^+$: 236. HR-MS $[M + H]^+$ calcd for $C_{12}H_{22}N_5^+$: 236.1870, found 236.1878.

4-(4-Cyclobutylpiperazin-1-yl)-6-isopropylpyrimidin-2-amine (11). A microwave vial charged with chloride **5** (453 mg, 2.64 mmol), 1-cyclobutylpiperazine (370 mg, 2.64 mmol), DIPEA (0.46 mL, 2.64 mmol) and dioxane (15 mL) was heated for 30 min at 150 °C under microwave irradiation. The reaction mixture was diluted with water (30 mL) and extracted with DCM (3 x 25 mL). The combined organic layers were dried over Na_2SO_4, filtered and concentrated *in vacuo*. Purification by flash chromatography (DCM:MeOH:TEA 100:0:0 to 90:9:1) gave the title compound as a white solid (428 mg, 59 %). Mp: 126.3-126.7 °C. ^1H NMR (500 MHz, $CDCl_3$) δ 5.81 (s, 1H), 4.77 (br, 2H), 3.60 (t, *J* = 5.0 Hz, 4H), 2.78 – 2.62 (m, 2H), 2.35 (t, *J* = 5.1 Hz, 4H), 2.09 – 2.01 (m, 2H), 1.95 – 1.85 (m, 2H), 1.79 – 1.63 (m, 2H), 1.21 (d, *J* = 6.8 Hz, 6H). ^{13}C NMR (126 MHz, $CDCl_3$) δ 175.1, 163.6, 162.3, 90.1, 60.3, 49.2, 43.7, 36.0, 27.2, 21.9, 14.4. HPLC-MS (basic mode): t_R = 4.2 min, purity: 97.7 %, $[M + H]^+$: 276. HR-MS $[M + H]^+$ calcd for $C_{15}H_{26}N_5^+$: 276.2183, found 276.2177.

4-(4-Cyclobutylpiperazin-1-yl)-6-methylpyrimidin-2-amine (12). To a solution of carbamate **9** (960 mg, 1.88 mmol) in dioxane (18 mL) was added HCl in dioxane (4N, 8.2 mL, 32.8 mmol). The reaction mixture was stirred for 2 h at rt. The reaction mixture was diluted with satd. aq. Na_2CO_3 (10 mL) and brine (40 mL) and extracted with DCM (3 x 30 mL). The combined organic layers were dried over Na_2SO_4, filtered and concentrated *in vacuo*. The resulting crude 4-methyl-6-(piperazin-1-yl)pyrimidin-2-amine (305 mg) was used for the next step without further purification. To a solution of crude amine intermediate in DCM (8 mL) was added cyclobutanone (140 µL, 1.88 mmol). After 10 min of stirring at rt, $NaBH(OAc)_3$ (446 mg, 2.36 mmol) was added and the resulting mixture was stirred at rt overnight. The reaction mixture was quenched with satd. aq. Na_2CO_3 solution (10 mL) and extracted with DCM (3 x 5 mL). The combined organic phases were dried over Na_2SO_4, filtered and concentrated *in vacuo*. The crude product was purified by flash chromatography (DCM:MeOH 10:0 to 9:1). The selected fractions were collected and the solvents were removed under reduced pressure. The residue was triturated with Et_2O to give the title compound as a white solid (155 mg, 33 % over two steps). Mp: 162.5-163.4 °C. ^1H NMR (500 MHz, $CDCl_3$) δ 5.79 (s, 1H), 4.78 (br, 2H), 3.65 – 3.46 (m, 4H), 2.70 (p, *J* = 7.9 Hz, 1H), 2.36 – 2.28 (m, 4H), 2.18 (s, 3H), 2.07 – 1.98 (m, 2H), 1.93 – 1.81 (m, 2H), 1.76 – 1.61 (m, 2H). ^{13}C NMR (126 MHz, $CDCl_3$) δ 166.2, 163.4, 162.5, 93.1, 60.2, 49.2 43.6, 27.2, 24.2, 14.4. HPLC-MS (basic mode): t_R = 3.8 min, purity: 97.7 %, $[M + H]^+$: 248. HR-MS $[M + H]^+$ calcd for $C_{13}H_{22}N_5^+$: 248.1870, found 248.1872.

***Tert*-butyl (1-(2-chloro-6-isopropylpyrimidin-4-yl)azetidin-3-yl)(methyl)carbamate (13).** To a solution of amine **7** (500 mg, 1.56 mmol) in DCM (20 mL) was added $SbCl_3$ (710 mg, 3.11 mmol) and *t*BuONO (0.72 mL, 6.22 mmol). The reaction mixture was stirred for 3 h at rt. Aq. Na_2CO_3 (1.0 M, 20 mL)

was added. The suspension was filtered. The filtrate was extracted with DCM (3 x 10 mL). The combined organic layers were dried over Na_2SO_4, filtered and concentrated *in vacuo*. Purification by flash chromatography (DCM:MeOH 10:0 to 9:1) gave the title compound as a yellow oil (202 mg, 40 %). ^{1}H NMR (250 MHz, $CDCl_3$) δ 5.91 (s, 1H), 5.03 (br, 1H), 4.30 (t, *J* = 8.8 Hz, 2H), 4.14 (dd, *J* = 9.2, 5.8 Hz, 2H), 2.93 (s, 3H), 2.90 – 2.73 (m, 1H), 1.47 (s, 9H), 1.24 (d, *J* = 6.9 Hz, 6H). HPLC-MS (acidic mode): t_R = 5.0 min, purity: 82.7 %, $[M + H]^+$: 341.

***Tert*-butyl 4-(2-chloro-6-isopropylpyrimidin-4-yl)piperazine-1-carboxylate (14).** To a solution of amine **8** (1.10 g, 3.42 mmol) in DCM (40 mL) was added $SbCl_3$ (1.56 g, 6.84 mmol) and *t*BuONO (1.57 mL, 13.7 mmol). The reaction mixture was stirred for 3 h at rt. Aq. Na_2CO_3 (1.0 M, 40 mL) was added. The suspension was filtered. The filtrate was extracted with DCM (3 x 20 mL). The combined organic layers were dried over Na_2SO_4, filtered and concentrated *in vacuo*. Purification by flash chromatography (DCM:MeOH 100:0 to 93:7) gave the title compound as a yellow oil (337 mg, 29 %). ^{1}H NMR (250 MHz, $CDCl_3$) δ 6.22 (s, 1H), 3.71 – 3.58 (m, 4H), 3.58 – 3.42 (m, 4H), 2.82 (hept, *J* = 6.9 Hz, 1H), 1.48 (s, 9H), 1.24 (d, *J* = 6.9 Hz, 6H). HPLC-MS (acidic mode): t_R = 5.3 min, purity: 91.5 %, $[M + H]^+$: 341.

***Tert*-butyl 4-(2-chloro-6-methylpyrimidin-4-yl)piperazine-1-carboxylate (15).** To a solution of amine **9** (2.00 g, 6.82 mmol) in DCM (40 mL) was added $SbCl_3$ (3.11 g, 13.6 mmol) and *i*PeONO (3.66 mL, 27.3 mmol). The reaction mixture was stirred for 3 h at rt. Aq. Na_2CO_3 (1.0 M, 50 mL) was added. The suspension was filtered. The filtrate was extracted with DCM (3 x 20 mL). The combined organic layers were dried over Na_2SO_4, filtered and concentrated *in vacuo*. Purification by flash chromatography (DCM:MeOH 20:0 to 19:1) gave the title compound as a yellow oil (496 mg, 23 %). ^{1}H NMR (250 MHz, $CDCl_3$) δ 6.25 (s, 1H), 3.69 – 3.58 (m, 4H), 3.57 – 3.45 (m, 4H), 2.35 (s, 3H), 1.48 (s, 9H). HPLC-MS (acidic mode): t_R = 4.5 min, purity: 98.9 %, $[M + H]^+$: 313.

1-(2-Chloro-6-isopropylpyrimidin-4-yl)-N,N-dimethylazetidin-3-amine (16). To a solution of carbamate **13** (200 mg, 0.59 mmol) in dioxane (5 mL) was added HCl in dioxane (4N, 1.47 mL, 5.87 mmol). The reaction mixture was stirred for 3 h at rt. The solvent was evaporated. The residue was diluted with aq. Na_2CO_3 (1.0 M, 10 mL) and extraction with DCM (3 x 8 mL) was performed. The combined organic phases were dried over Na_2SO_4, filtered and concentrated *in vacuo*. The resulting crude 1-(2-chloro-6-isopropylpyrimidin-4-yl)-N-methylazetidin-3-amine (131 mg) was used for the next step without further purification. To a solution of crude amine intermediate in DCM (5 mL) was added formalin (37 %, 46 µL, 0.62 mmol). After 10 min of stirring at rt, $NaBH(OAc)_3$ (220 mg, 1.04 mmol) was added and the resulting mixture was stirred at rt overnight. The reaction mixture was quenched with satd. aq. $NaHCO_3$ (10 mL) and extracted with DCM (3 x 5 mL). The combined organic phases were dried over Na_2SO_4, filtered and concentrated *in vacuo*. Purification by flash chromatography (DCM:MeOH 20:0 to 19:1) gave the title compound as a colourless oil (110 mg, 73 % over two steps). ^{1}H NMR (250 MHz, $CDCl_3$) δ 5.89 (s, 1H), 4.12 (t, *J* = 8.0 Hz, 2H), 4.02 – 3.87 (m, 2H), 3.33 – 3.19 (m, 1H), 2.79 (hept, *J* = 7.1 Hz, 1H), 2.21 (s, 6H), 1.22 (d, *J* = 6.9 Hz, 6H). HPLC-MS (acidic mode): t_R = 2.5 min, purity: 98.8 %, $[M + H]^+$: 255.

2-Chloro-4-(4-cyclobutylpiperazin-1-yl)-6-isopropylpyrimidine (17). To a solution of carbamate **14** (330 mg, 0.97 mmol) in dioxane (5 mL) was added HCl in dioxane (4N, 3.60 mL, 14.4 mmol). The reaction mixture was stirred for 2 h at rt. The solvent was evaporated. The residue was diluted with aq. Na_2CO_3 (1.0 M, 10 mL) and extracted with DCM (3 x 8 mL). The combined organic phases were dried over Na_2SO_4, filtered and concentrated *in vacuo*. The resulting crude 2-chloro-4-isopropyl-6-(piperazine-1-yl)pyrimidin (234 mg) was used for the next step without further purification. To a solution of crude amine intermediate in DCM (10 mL) was added cyclobutanone (85 µL, 1.14 mmol). After 10 min of stirring at rt, $NaBH(OAc)_3$ (301 mg, 1.42 mmol) was added and the resulting mixture was stirred at rt overnight. The reaction mixture

was quenched with satd. aq. $NaHCO_3$ (20 mL) and extracted with DCM (3 x 10 mL). The combined organic phases were dried over Na_2SO_4, filtered and concentrated *in vacuo*. Purification by flash chromatography (DCM:MeOH 20:0 to 19:1) gave the title compound as a colourless oil (200 mg, 70 % over two steps). ^1H NMR (250 MHz, $CDCl_3$) δ 6.21 (s, 1H), 3.68 (app. s, 4H), 2.91 – 2.69 (m, 2H), 2.48 – 2.34 (m, 4H), 2.14 – 2.00 (m, 2H), 2.00 – 1.84 (m, 2H), 1.83 – 1.62 (m, 2H), 1.24 (d, *J* = 6.9 Hz, 6H). HPLC-MS (acidic mode): t_R = 3.2 min, purity: >99 %, $[M + H]^+$: 295.

2-Chloro-4-(4-cyclobutylpiperazin-1-yl)-6-methylpyrimidine (18). To a solution of carbamate **15** (490 mg, 1.57 mmol) in dioxane (10 mL) was added HCl in dioxane (4N, 4.50 mL, 18.0 mmol). The reaction mixture was stirred at rt overnight. The solvent was evaporated. The residue was diluted with aq. Na_2CO_3 (1.0 M, 10 mL) and extracted with DCM (3 x 10 mL). The combined organic phases were dried over Na_2SO_4, filtered and concentrated *in vacuo*. The resulting crude 2-chloro-4-methyl-6-(piperazine-1-yl)pyrimidin (232 mg) was used for the next step without further purification. To a solution of crude amine intermediate in DCM (8 mL) was added cyclobutanone (95 µL, 1.27 mmol). After 10 min of stirring at rt, $NaBH(OAc)_3$ (336 mg, 1.59 mmol) was added and the resulting mixture was stirred at rt overnight. The reaction mixture was quenched with satd. aq. Na_2CO_3 (15 mL) and extracted with DCM (3 x 10 mL). The combined organic phases were dried over Na_2SO_4, filtered and concentrated *in vacuo*. Purification by flash chromatography (DCM:MeOH 20:0 to 19:1) gave the title compound as an off-white solid (236 mg, 56 % over two steps). ^1H NMR (250 MHz, $CDCl_3$) δ 6.23 (s, 1H), 3.65 (app. s, 4H), 2.74 (p, *J* = 7.6 Hz, 1H), 2.42 – 2.34 (m, 4H), 2.33 (s, 3H), 2.13 – 1.96 (m, 2H), 1.96 – 1.79 (m, 2H), 1.79 – 1.61 (m, 2H). HPLC-MS (acidic mode): t_R = 2.1 min, purity: >99 %, $[M + H]^+$: 267.

4-(4-Cyclobutylpiperazin-1-yl)-6-methyl-2-((trimethylsilyl)ethynyl)pyrimidine (19). A microwave vial was charged with chloride **18** (72 mg, 0.27 mmol), DME (2 mL), TEA (0.5 mL) and ethynyltrimethylsilane (76 µL, 0.54 mmol). The mixture was degassed with N_2 for 10 min. $Pd(dppf)Cl_2$ (11 mg, 0.013 mmol) and CuI (5.1 mg, 0.027 mmol) were added. The reaction mixture was heated for 1 h at 100 °C under microwave irradiation. The reaction mixture was diluted with water (10 mL) and extracted with DCM (3 x 8 mL). The combined organic layers were dried over Na_2SO_4, filtered and concentrated *in vacuo*. Purification by flash chromatography (DCM:MeOH 20:0 to 19:1) gave the title compound as a colourless oil (48 mg, 54 %). ^1H NMR (500 MHz, $CDCl_3$) δ 6.28 (s, 1H), 3.65 (br, 4H), 2.72 (p, *J* = 7.8 Hz, 1H), 2.36 (t, *J* = 5.2 Hz, 4H), 2.34 (s, 3H), 2.10 – 2.00 (m, 2H), 1.96 – 1.83 (m, 2H), 1.78 – 1.63 (m, 2H), 0.25 (s, 9H). HPLC-MS (acidic mode): t_R = 3.5 min, purity: 96.4 %, $[M + H]^+$: 329.

4-(4-Cyclobutylpiperazin-1-yl)-2-ethynyl-6-methylpyrimidine (20). To a solution of alkyne **19** (48 mg, 0.15 mmol) in MeOH (2 mL) was added K_2CO_3 (20 mg, 0.15 mmol). The reaction mixture was stirred for 1 h at rt. The solvent was evaporated. The residue was diluted with water (10 mL) and extracted with DCM (3 x 8 mL). The combined organic layers were dried over Na_2SO_4, filtered and concentrated *in vacuo*. Purification by flash chromatography (DCM:MeOH 98:2 to 94:6) gave the title compound as a colourless oil (27 mg, 72 %). ^1H NMR (500 MHz, $CDCl_3$) δ 6.29 (s, 1H), 3.62 (br, 4H), 2.91 (s, 1H), 2.70 (p, *J* = 7.9 Hz, 1H), 2.36 – 2.32 (m, 4H), 2.31 (s, 3H), 2.07 – 1.97 (m, 2H), 1.92 – 1.80 (m, 2H), 1.76 – 1.59 (m, 2H). ^{13}C NMR (126 MHz, $CDCl_3$) δ 165.6, 161.9, 150.8, 101.2, 82.8, 73.1, 60.1, 49.0, 43.6, 27.1, 24.3, 14.3. HPLC-MS (basic mode): t_R = 4.3 min, purity: 96.2 %, $[M + H]^+$: 257. HR-MS $[M + H]^+$ calcd for $C_{15}H_{21}N_4^+$: 257.1761, found 257.1766.

1-(6-Isopropyl-2-vinylpyrimidin-4-yl)-N,N-dimethylazetidin-3-amine (21). To a solution of chloride **16** (105 mg, 0.41 mmol) and vinylboronic acid MIDA ester (75 mg, 0.41 mmol) in DME (2.5 mL) was added Na_2CO_3 (87 mg, 0.82 mmol) in H_2O (0.8 mL). The mixture was degassed with N_2 for 10 min and $Pd(PPh_3)_4$ (23.8 mg, 0.021 mmol) in DME (0.5 mL) was added. The reaction mixture was heated for 1 h at 120 °C under

microwave irradiation. The reaction mixture was diluted with water (20 mL) and extracted with DCM (3 x 15 mL). The combined organic layers were dried over Na_2SO_4, filtered and concentrated *in vacuo*. Purification by flash chromatography (DCM:MeOH 100:0 to 93:7) gave the title compound as a yellow oil (63 mg, 62 %). ^{1}H NMR (500 MHz, $CDCl_3$) δ 6.70 (dd, *J* = 17.2, 10.4 Hz, 1H), 6.49 (dd, *J* = 17.2, 2.2 Hz, 1H), 5.90 (s, 1H), 5.56 (dd, *J* = 10.4, 2.2 Hz, 1H), 4.10 (t, *J* = 7.8 Hz, 2H), 3.90 (dd, *J* = 8.3, 5.4 Hz, 2H), 3.29 – 3.19 (m, 1H), 2.81 (hept, *J* = 6.9 Hz, 1H), 2.21 (s, 6H), 1.23 (d, *J* = 6.9 Hz, 6H). ^{13}C NMR (126 MHz, $CDCl_3$) δ 173.8, 163.8, 163.2, 137.6, 122.3, 96.2, 56.6, 54.1, 42.0, 35.9, 22.0. HPLC-MS (basic mode): t_R = 4.7 min, purity: >99 %, $[M + H]^+$: 257. HR-MS $[M + H]^+$ calcd for $C_{14}H_{23}N_4^+$: 247.1917, found 247.1918.

4-(4-Cyclobutylpiperazin-1-yl)-6-isopropyl-2-vinylpyrimidine (22). To a solution of chloride **17** (195 mg, 0.66 mmol) and vinylboronic acid MIDA ester (121 mg, 0.66 mmol) in DME (4 mL) was added Na_2CO_3 (140 mg, 1.32 mmol) in H_2O (1 mL). The mixture was degassed with N_2 for 10 min and $Pd(PPh_3)_4$ (76 mg, 0.066 mmol) was added. The reaction mixture was heated for 30 min at 120 °C under microwave irradiation. The reaction mixture was diluted with water (20 mL) and extracted with DCM (3 x 15 mL). The combined organic layers were dried over Na_2SO_4, filtered and concentrated *in vacuo*. Purification by flash chromatography (DCM:MeOH 20:0 to 19:1) gave the title compound as a yellow oil (40 mg, 21 %). ^{1}H NMR (500 MHz, $CDCl_3$) δ 6.71 (dd, *J* = 17.2, 10.4 Hz, 1H), 6.49 (dd, *J* = 17.2, 2.2 Hz, 1H), 6.21 (s, 1H), 5.57 (dd, *J* = 10.4, 2.2 Hz, 1H), 3.79 – 3.60 (m, 4H), 2.90 – 2.70 (m, 2H), 2.48 – 2.34 (m, 4H), 2.13 – 2.01 (m, 2H), 2.00 – 1.87 (m, 2H), 1.80 – 1.65 (m, 2H), 1.25 (d, *J* = 6.9 Hz, 6H). ^{13}C NMR (126 MHz, $CDCl_3$) δ 174.7, 163.1, 162.6, 137.8, 122.2, 96.9, 60.3, 49.2, 43.6, 36.2, 27.1, 22.1, 14.4. HPLC-MS (acidic mode): t_R = 1.8 min, purity: 98.6 %, $[M + H]^+$: 287. HR-MS $[M + H]^+$ calcd for $C_{17}H_{27}N_4^+$: 287.2222, found 287.2230.

4-(4-Cyclobutylpiperazin-1-yl)-6-methyl-2-vinylpyrimidine (23). To a solution of chloride **18** (80 mg, 0.30 mmol) and vinylboronic acid MIDA ester (58 mg, 0.32 mmol) in DME (1.6 mL) was added Na_2CO_3 (64 mg, 0.60 mmol) in H_2O (0.4 mL). The mixture was degassed with N_2 for 10 min and $Pd(PPh_3)_4$ (35 mg, 0.030 mmol) was added. The reaction mixture was heated for 1 h at 120 °C under microwave irradiation. The reaction mixture was diluted with water (10 mL) and extracted with DCM (3 x 8 mL). The combined organic layers were dried over Na_2SO_4, filtered and concentrated *in vacuo*. Purification by flash chromatography (DCM:MeOH 100:0 to 94:6) gave the title compound as a yellow oil (20 mg, 26 %). ^{1}H NMR (500 MHz, $CDCl_3$) δ 6.69 (dd, *J* = 17.3, 10.4 Hz, 1H), 6.48 (dd, *J* = 17.3, 2.1 Hz, 1H), 6.23 (s, 1H), 5.58 (dd, *J* = 10.4, 2.1 Hz, 1H), 3.68 (t, *J* = 5.2 Hz, 4H), 2.74 (p, *J* = 8.0 Hz, 1H), 2.43 – 2.37 (m, 4H), 2.36 (s, 3H), 2.11 – 2.01 (m, 2H), 1.97 – 1.86 (m, 2H), 1.79 – 1.64 (m, 2H). ^{13}C NMR (126 MHz, $CDCl_3$) δ 165.5, 163.2, 162.3, 137.5, 122.4, 99.9, 60.3, 49.2, 43.5, 27.2, 24.5, 14.4. HPLC-MS basic mode): t_R = 4.5 min, purity: 96.9 %, $[M + H]^+$: 259. HR-MS $[M + H]^+$ calcd for $C_{15}H_{23}N_4^+$: 259.1917, found 259.1919.

6-Isopropyl-2-methylpyrimidin-4(3*H*)-one (26). To an ice-cold solution of acetamidine hydrochloride **24** (2.08 g, 22.0 mmol) and methyl 4-methyl-3-oxopentanoate (2.88 g, 20.0 mmol) in MeOH (25 mL) was added NaOMe (3.24 g, 60.0 mmol) in portions. The reaction mixture was stirred at rt overnight. The reaction mixture was concentrated to half the volume and diluted with water (50 mL). The pH of the filtrate was adjusted to 4 with aq. HCl (3.0 M). The mixture was extracted with DCM (3 x 40 mL). The combined organic layers were dried over Na_2SO_4, filtered and concentrated *in vacuo*. Purification by flash chromatography (DCM:MeOH 10:0 to 9:1) gave the title compound as a white solid (2.77 g, 91 %). ^{1}H NMR (500 MHz, $CDCl_3$) δ 13.36 (br, 1H), 6.18 (s, 1H), 2.75 (hept, *J* = 6.8 Hz, 1H), 2.47 (s, 3H), 1.22 (d, *J* = 6.9 Hz, 6H). HPLC-MS (acidic mode): t_R = 1.9 min, purity: >99 %, $[M + H]^+$: 153.

2-Ethyl-6-isopropylpyrimidin-4(3*H*)-one (27). To an ice-cold solution of propionamidine hydrochloride **25** (1.00 g, 9.21 mmol) and methyl 4-methyl-3-oxopentanoate (1.21 g, 8.37 mmol) in MeOH (10 mL) was added NaOMe (1.36 g, 25.1 mmol) in portions. The reaction mixture was stirred at rt overnight.

The reaction mixture was concentrated to half the volume and diluted with water (20 mL). The pH of the filtrate was adjusted to 4 with aq. HCl (3.0 M). The mixture was extracted with DCM (3 x 20 mL). The combined organic layers were dried over Na_2SO_4, filtered and concentrated *in vacuo*. Purification by flash chromatography (DCM:MeOH 10:0 to 9:1) gave the title compound as a white solid (1.35 g, 97 %). ^{1}H NMR (500 MHz, $CDCl_3$) δ 13.28 (s, 1H), 6.18 (s, 1H), 2.88 – 2.61 (m, 3H), 1.33 (t, *J* = 7.6 Hz, 3H), 1.21 (d, *J* = 6.9 Hz, 6H). HPLC-MS (acidic mode): t_R = 1.9 min, purity: >99 %, $[M + H]^+$: 167.

4-Chloro-6-isopropyl-2-methylpyrimidine (28). Pyrimidin-4(3*H*)-one **26** (2.76 g, 18.1 mmol) was dissolved in $POCl_3$ (30 mL, 0.32 mol). The mixture was heated to reflux for 3 h. The solvent was removed under reduced pressure. Ice (60 g) was carefully added to the residue. The pH of the mixture was adjusted to 9-10 with aq. NaOH (2.5 M). The mixture was extracted with DCM (3 x 60 mL). The combined organic phases were dried over Na_2SO_4, filtered and concentrated *in vacuo*. Purification by flash chromatography (DCM:MeOH 20:0 to 19:1) gave the title compound as a yellow oil (2.70 g, 87 %). ^{1}H NMR (500 MHz, $CDCl_3$) δ 7.03 (s, 1H), 2.95 (hept, *J* = 6.9 Hz, 1H), 2.68 (s, 3H), 1.28 (d, *J* = 6.9 Hz, 6H). HPLC-MS (acidic mode): t_R = 4.1 min, purity: >99 %, $[M + H]^+$: 171.

4-Chloro-2-ethyl-6-isopropylpyrimidine (29). Pyrimidin-4(3*H*)-one **27** (1.34 g, 8.06 mmol) was dissolved in $POCl_3$ (10 mL, 107 mmol). The mixture was heated to reflux for 3 h. The solvent was removed under reduced pressure. Ice (30 g) was carefully added to the residue. The pH of the mixture was adjusted to 9-10 with aq. NaOH (2.5 M). The mixture was extracted with DCM (3 x 20 mL). The combined organic phases were dried over Na_2SO_4, filtered and concentrated *in vacuo*. Purification by flash chromatography (DCM:MeOH 20:0 to 19:1) gave the title compound as a yellow oil (1.27 g, 86 %). ^{1}H NMR (500 MHz, $CDCl_3$) δ 7.03 (s, 1H), 3.07 – 2.85 (m, 3H), 1.34 (t, *J* = 7.6 Hz, 3H), 1.29 (d, *J* = 6.9 Hz, 6H). HPLC-MS (acidic mode): t_R = 5.0 min, purity: >99 %, $[M + H]^+$: 185.

***Tert*-butyl (1-(6-isopropyl-2-methylpyrimidin-4-yl)azetidin-3-yl)(methyl)carbamate (30).** A microwave vial charged with chloride **28** (853 mg, 5.00 mmol), *tert*-butyl azetidin-3-yl(methyl)carbamate hydrochloride (1.11 g, 5.00 mmol), DIPEA (1.75 mL, 10.0 mmol) and dioxane (30 mL) was heated for 30 min at 150 °C under microwave irradiation. The reaction mixture was diluted with water (60 mL) and extracted with DCM (3 x 40 mL). The combined organic layers were dried over Na_2SO_4, filtered and concentrated *in vacuo*. Purification by flash chromatography (DCM:MeOH 10:0 to 9:1) gave the title compound as an off-white solid (602 mg, 38 %). ^{1}H NMR (250 MHz, $CDCl_3$) δ 5.87 (s, 1H), 5.04 (br, 1H), 4.26 (t, *J* = 8.7 Hz, 2H), 4.06 (dd, *J* = 9.4, 5.7 Hz, 2H), 2.94 (s, 3H), 2.81 (hept, *J* = 7.0 Hz, 1H), 2.51 (s, 3H), 1.47 (s, 9H), 1.23 (d, *J* = 6.9 Hz, 6H). HPLC-MS (acidic mode): t_R = 3.2 min, purity: >99 %, $[M + H]^+$: 321.

***Tert*-butyl (1-(2-ethyl-6-isopropylpyrimidin-4-yl)azetidin-3-yl)(methyl)carbamate (31).** A microwave vial charged with chloride **29** (739 mg, 4.00 mmol), *tert*-butyl azetidin-3-yl(methyl)carbamate hydrochloride (891 mg, 4.00 mmol), DIPEA (1.40 mL, 8.00 mmol) and dioxane (20 mL) was heated for 30 min at 150 °C under microwave irradiation. The reaction mixture was diluted with water (40 mL) and extracted with DCM (3 x 30 mL). The combined organic layers were dried over Na_2SO_4, filtered and concentrated *in vacuo*. Purification by flash chromatography (DCM:MeOH 10:0 to 9:1) gave the title compound as a colourless oil (460 mg, 34 %). ^{1}H NMR (250 MHz, $CDCl_3$) δ 5.87 (s, 1H), 5.03 (br, 1H), 4.26 (t, *J* = 8.7 Hz, 2H), 4.07 (dd, *J* = 9.4, 5.8 Hz, 2H), 2.94 (s, 3H), 2.90 – 2.68 (m, 3H), 1.46 (s, 9H), 1.28 (t, *J* = 7.6 Hz, 3H), 1.23 (d, *J* = 6.9 Hz, 6H)). HPLC-MS (acidic mode): t_R = 4.3 min, purity: >99 %, $[M + H]^+$: 335.

1-(6-Isopropyl-2-methylpyrimidin-4-yl)-N-methylazetidin-3-amine (32). To a solution of carboxylate **30** (590 mg, 1.84 mmol) in dioxane (10 mL) was added HCl in dioxane (4N, 4.60 mL, 18.4 mmol). The reaction mixture was stirred for 3 h at rt. The solvent was removed under reduced pressure. The residue

was mixed with aq. Na_2CO_3 (1.0 M, 40 mL) and extracted with DCM (3 x 20 mL). The combined organic layers were dried over Na_2SO_4, filtered and concentrated *in vacuo*. Purification by flash chromatography (DCM:MeOH 10:0 to 9:1) gave the title compound as a colourless oil (314 mg, 77 %). ^1H NMR (500 MHz, $CDCl_3$) δ 5.84 (s, 1H), 4.28 – 4.20 (m, 2H), 3.76 (dd, *J* = 8.6, 4.9 Hz, 2H), 3.74 – 3.68 (m, 1H), 2.80 (hept, *J* = 6.9 Hz, 1H), 2.49 (s, 3H), 2.43 (s, 3H), 1.22 (d, *J* = 6.9 Hz, 6H). HPLC-MS (basic mode): t_R = 3.2 min, purity: 98.1 %, $[M + H]^+$: 221.

1-(2-Ethyl-6-isopropylpyrimidin-4-yl)-N-methylazetidin-3-amine (33). To a solution of carboxylate **31** (450 mg, 1.34 mmol) in dioxane (8 mL) was added HCl in dioxane (4N, 4.60 mL, 18.4 mmol). The reaction mixture was stirred at rt overnight. The solvent was removed under reduced pressure. The residue was mixed with aq. Na_2CO_3 (1.0 M, 20 mL) and extracted with DCM (3 x 15 mL). The combined organic layers were dried over Na_2SO_4, filtered and concentrated *in vacuo*. Purification by flash chromatography (DCM:MeOH 10:0 to 9:1) gave the title compound as a colourless oil (283 mg, 90 %). ^1H NMR (500 MHz, $CDCl_3$) δ 5.85 (s, 1H), 4.28 – 4.20 (m, 2H), 3.77 (dd, *J* = 8.6, 4.9 Hz, 2H), 3.74 – 3.69 (m, 1H), 2.82 (hept, *J* = 6.9 Hz, 1H), 2.73 (q, *J* = 7.6 Hz, 2H), 2.43 (s, 3H), 1.28 (t, *J* = 7.6 Hz, 3H), 1.22 (d, *J* = 6.9 Hz, 6H). HPLC-MS (basic mode): t_R = 3.6 min, purity: 95.4 %, $[M + H]^+$: 235.

1-(6-Isopropyl-2-methylpyrimidin-4-yl)-N,N-dimethylazetidin-3-amine (34). To a solution of carbamate **32** (200 mg, 0.91 mmol) in dioxane (5 mL) was added formalin (37 %, 102 µL, 1.34 mmol). After 10 min of stirring at rt, $NaBH(OAc)_3$ (481 mg, 2.27 mmol) was added and the resulting mixture was stirred at rt overnight. The reaction mixture was quenched with satd. aq. Na_2CO_3 (20 mL) and extracted with DCM (3 x 10 mL). The combined organic phases were dried over Na_2SO_4, filtered and concentrated *in vacuo*. Purification by flash chromatography (DCM:MeOH 20:0 to 19:1) gave the title compound as a colourless oil (175 mg, 82 %). ^1H NMR (500 MHz, $CDCl_3$) δ 5.84 (s, 1H), 4.09 (t, *J* = 7.8 Hz, 2H), 3.88 (dd, *J* = 8.3, 5.4 Hz, 2H), 3.22 (tt, *J* = 7.0, 5.2 Hz, 1H), 2.79 (hept, *J* = 6.9 Hz, 1H), 2.49 (s, 3H), 2.20 (s, 6H), 1.21 (d, *J* = 6.9 Hz, 6H). ^{13}C NMR (126 MHz, $CDCl_3$) δ 173.7, 166.9, 163.8, 94.7, 56.5, 54.2, 42.0, 35.8, 26.2, 22.0. HPLC-MS (basic mode): t_R = 3.5 min, purity: >99 %, $[M + H]^+$: 235. HR-MS $[M + H]^+$ calcd for $C_{13}H_{23}N_4^+$: 235.1917, found 235.1924.

1-(2-Ethyl-6-isopropylpyrimidin-4-yl)-N,N-dimethylazetidin-3-amine (35). To a solution of carbamate **33** (170 mg, 0.72 mmol) in dioxane (5 mL) was added formalin (37 %, 81 µL, 1.09 mmol). After 10 min of stirring at rt, $NaBH(OAc)_3$ (384 mg, 1.81 mmol) was added and the resulting mixture was stirred at rt overnight. The reaction mixture was quenched with satd. aq. Na_2CO_3 (20 mL) and extracted with DCM (3 x 10 mL). The combined organic phases were dried over Na_2SO_4, filtered and concentrated *in vacuo*. Purification by flash chromatography (DCM:MeOH 20:0 to 19:1) gave the title compound as a colourless oil (148 mg, 82 %). ^1H NMR (500 MHz, $CDCl_3$) δ 5.85 (s, 1H), 4.09 (t, *J* = 7.8 Hz, 2H), 3.88 (dd, *J* = 8.3, 5.3 Hz, 2H), 3.22 (tt, *J* = 7.0, 5.3 Hz, 1H), 2.81 (hept, *J* = 6.9 Hz, 1H), 2.73 (q, *J* = 7.6 Hz, 2H), 2.21 (s, 6H), 1.27 (t, *J* = 7.6 Hz, 3H), 1.22 (d, *J* = 6.9 Hz, 6H). ^{13}C NMR (126 MHz, $CDCl_3$) δ 173.6, 171.1, 164.0, 94.8, 56.6, 54.2, 42.0, 35.8, 32.8, 22.0, 13.2. HPLC-MS (basic mode): t_R = 4.0 min, purity: >99 %, $[M + H]^+$: 249. HR-MS $[M + H]^+$ calcd for $C_{14}H_{25}N_4^+$: 249.2074, found 249.2080.

4-(4-Cyclobutylpiperazin-1-yl)-6-isopropyl-2-methylpyrimidine (36). A microwave vial charged with chloride **28** (213 mg, 1.25 mmol), 1-cyclobutylpiperazine (175 mg, 1.25 mmol), DIPEA (218 µL, 1.25 mmol) and dioxane (10 mL) was heated for 30 min at 150 °C under microwave irradiation. The reaction mixture was diluted with water (20 mL) and extracted with DCM (3 x 15 mL). The combined organic layers were dried over Na_2SO_4, filtered and concentrated *in vacuo*. Purification by flash chromatography (DCM:MeOH 10:0 to 9:1) gave the title compound as a colourless oil (123 mg, 36 %). ^1H NMR (500 MHz, $CDCl_3$) δ 6.15 (s, 1H), 3.70 – 3.60 (m, 4H), 2.86 – 2.70 (m, 2H), 2.48 (s, 3H), 2.43 – 2.34 (m, 4H), 2.10 – 2.01 (m,

2H), 1.97 – 1.86 (m, 2H), 1.79 – 1.65 (m, 2H), 1.23 (d, *J* = 6.9 Hz, 6H). ^{13}C NMR (126 MHz, $CDCl_3$) δ 174.5, 166.8, 162.7, 95.4, 60.3, 49.2, 43.6, 36.1, 27.1, 26.2, 22.1, 14.4. HPLC-MS (basic mode): t_R = 4.7 min, purity: 96.3 %, [M + H]$^+$: 275. HR-MS [M + H]$^+$ calcd for $C_{16}H_{27}N_4^+$: 275.2230, found 275.2229.

4-(4-Cyclobutylpiperazin-1-yl)-2-ethyl-6-isopropylpyrimidine (37). A microwave vial charged with chloride **29** (277 mg, 1.50 mmol), 1-cyclobutylpiperazine (210 mg, 1.50 mmol), DIPEA (262 µL, 1.50 mmol) and dioxane (8 mL) was heated for 30 min at 150 °C under microwave irradiation. The reaction mixture was diluted with water (20 mL) and extracted with DCM (3 x 15 mL). The combined organic layers were dried over Na_2SO_4, filtered and concentrated *in vacuo*. Purification by flash chromatography (DCM:MeOH 10:0 to 9:1) gave the title compound as a colourless oil (220 mg, 51 %). ^{1}H NMR (500 MHz, $CDCl_3$) δ 6.16 (s, 1H), 3.72 – 3.60 (m, 4H), 2.86 – 2.69 (m, 4H), 2.40 (s, 4H), 2.10 – 2.01 (m, 2H), 1.97 – 1.86 (m, 2H), 1.79 – 1.64 (m, 2H), 1.28 (t, J = 7.6 Hz, 3H), 1.23 (d, J = 6.9 Hz, 6H). ^{13}C NMR (126 MHz, $CDCl_3$) δ 174.5, 170.9, 162.8, 95.4, 60.3, 49.2, 43.6, 36.2, 32.7, 27.2, 22.1, 14.4, 12.8. HPLC-MS (basic mode): t_R = 5.3 min, purity: 98.6 %, [M + H]$^+$: 289. HR-MS [M + H]$^+$ calcd for $C_{17}H_{29}N_4^+$: 289.2384, found 289.2387.

2-Chloro-N-(3-(4-cyclobutylpiperazin-1-yl)phenyl)acetamide (43). To a solution of aniline **42** (46 mg, 0.20 mmol) and TEA (33 µL, 0.24 mmol) in DCM (2 mL) was added 2-chloroacetyl chloride (17 µL, 0.22 mmol). The reaction mixture was stirred for 1 h at rt. The reaction mixture was diluted with satd. aq. Na_2CO_3 (10 mL) and extracted with DCM (3 x 5 mL). The combined organic layers were dried over Na_2SO_4, filtered and concentrated *in vacuo*. Purification by flash chromatography (DCM:MeOH 20:0 to 19:1) gave the title compound as a white solid (42 mg, 68 %). Mp: 136.4-137.0 °C. ^{1}H NMR (500 MHz, $CDCl_3$) δ 8.18 (s, 1H), 7.23 (t, *J* = 2.3 Hz, 1H), 7.21 (t, *J* = 8.2 Hz, 1H), 6.91 (dd, *J* = 8.0, 1.9 Hz, 1H), 6.72 (dd, *J* = 8.4, 2.4 Hz, 1H), 4.16 (s, 2H), 3.27 – 3.16 (m, 4H), 2.77 (p, *J* = 7.9 Hz, 1H), 2.47 (t, *J* = 5.0 Hz, 4H), 2.10 – 2.01 (m, 2H), 1.97 – 1.86 (m, 2H), 1.78 – 1.64 (m, 2H). ^{13}C NMR (126 MHz, $CDCl_3$) δ 163.8, 152.1, 137.6, 129.7, 112.7, 111.1, 107.6, 60.3, 49.4, 48.6, 43.1, 27.1, 14.4. HPLC-MS (acidic mode): t_R = 2.7 min, purity: >99 %, [M + H]$^+$: 308. HR-MS [M + H]$^+$ calcd for $C_{16}H_{23}ClN_3O^+$: 308.1524, found 308.1524.

Characterization of 44

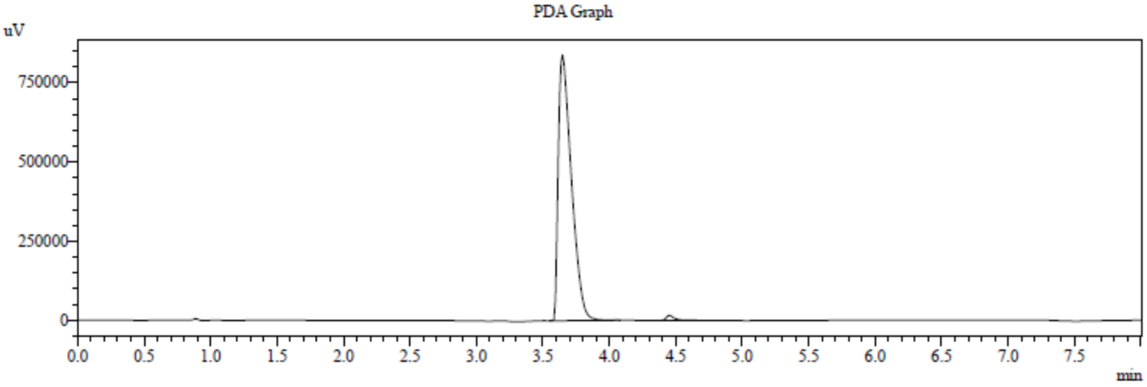

PDA Ch1 254nm 4nm

Peak#	Name	Ret. Time	Area	Area %
1		3.642	5784226	98.866
2		4.446	66330	1.134

MS Spectrum Graph

#:1 Ret.Time:Averaged 3.730-3.750(Scan#:374-376)
Mass Peaks:4 Base Peak:274.10(748303) Polarity:Pos Segment1 - Event1

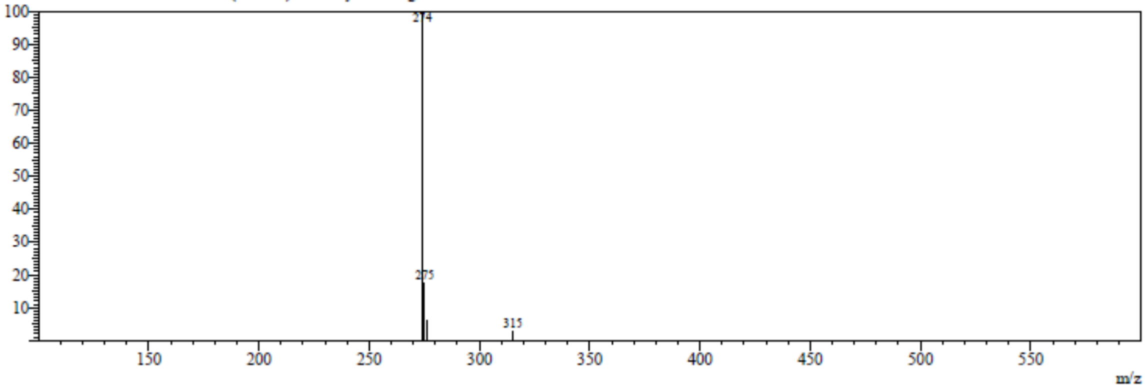

MS Spectrum Table

#:1 Ret.Time:
BG Mode:Calc 3.590<->4.090(360<->410)
Mass Peaks:4 Base Peak:274.10(748303) Polarity:Pos Segment1 - Event1

#	m/z	Abs.Inten.	Rel.Inten.	Charge	Polarity	Monoisotopic	#	m/z	Abs.Inten.	Rel.Inten.	Charge	Polarity	Monoisotopic
1	274.10	748303	100.00				3	276.05	45329	6.06			
2	275.10	130597	17.45				4	315.10	21508	2.87			

Figure S3. HPLC-MS chromatogram of compound **44.**

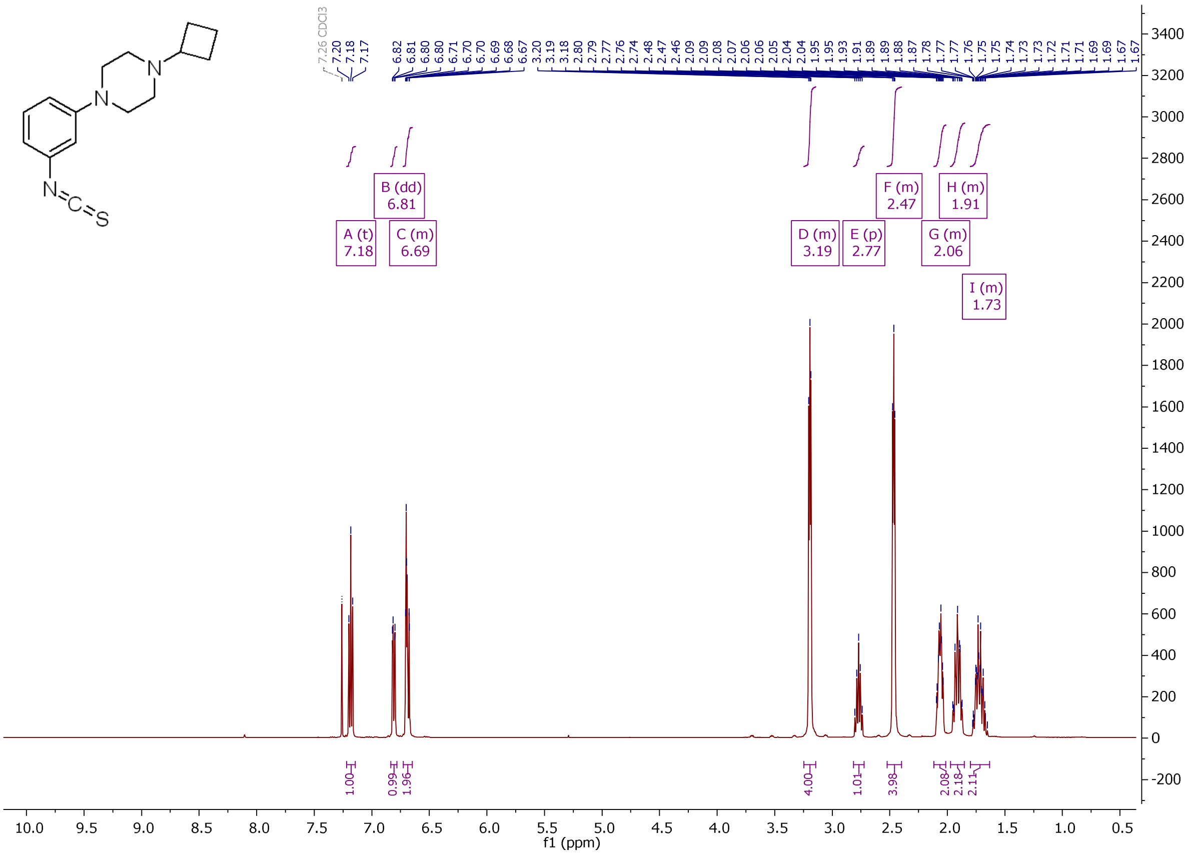

Figure S4. ^{1}H NMR spectrum of compound **44** in $CDCl_3$.

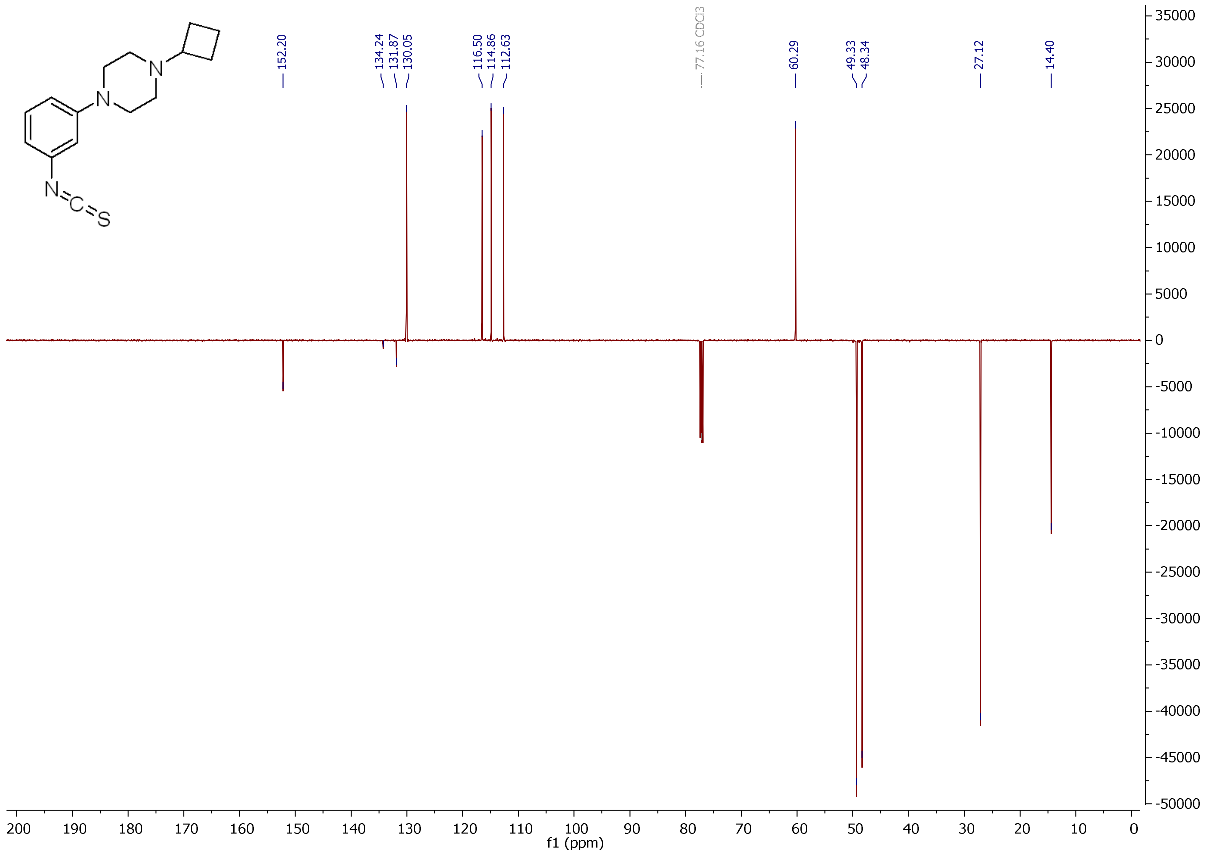

Figure S5. ^{13}C NMR spectrum of compound **44** in $CDCl_3$.

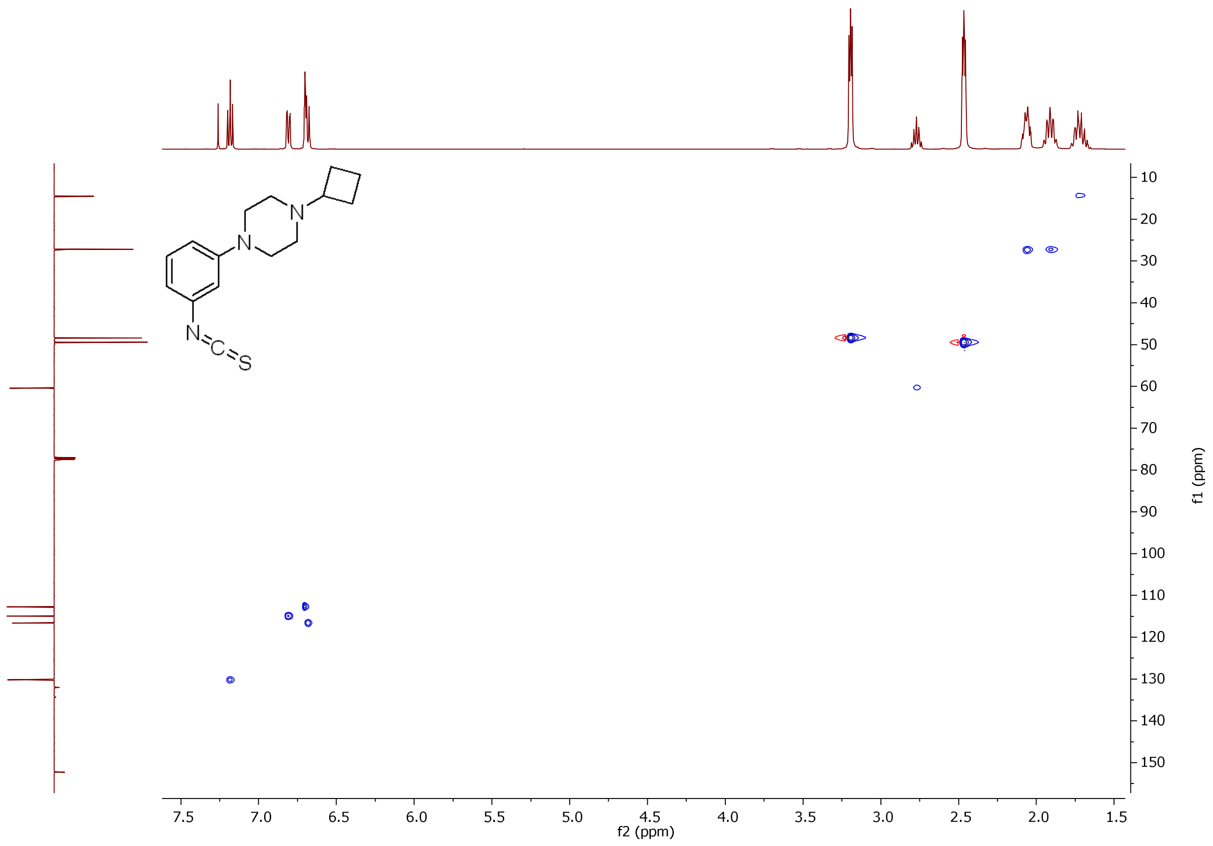

Figure S6. HSQC NMR spectrum of compound **44** in $CDCl_3$.

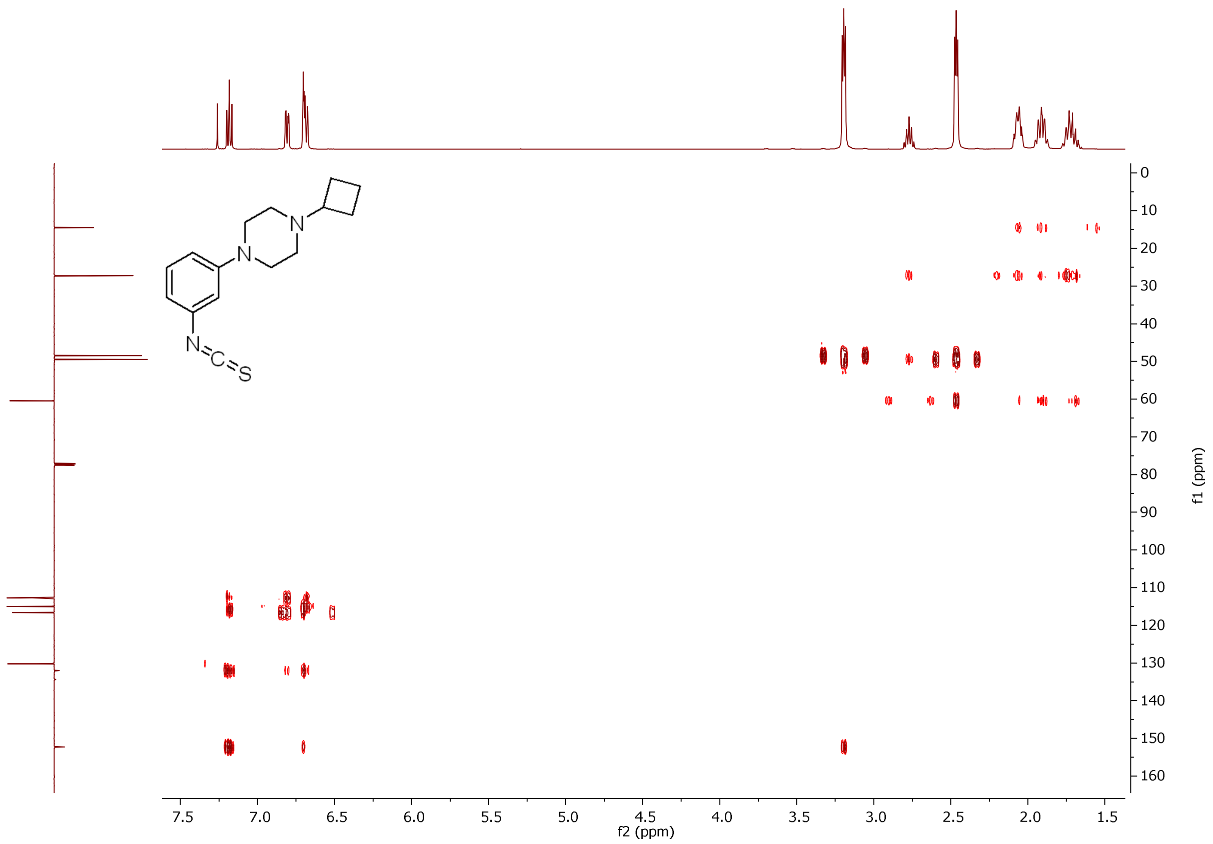

Figure S7. HMBC NMR spectrum of compound **44** in $CDCl_3$.

PDA Graph

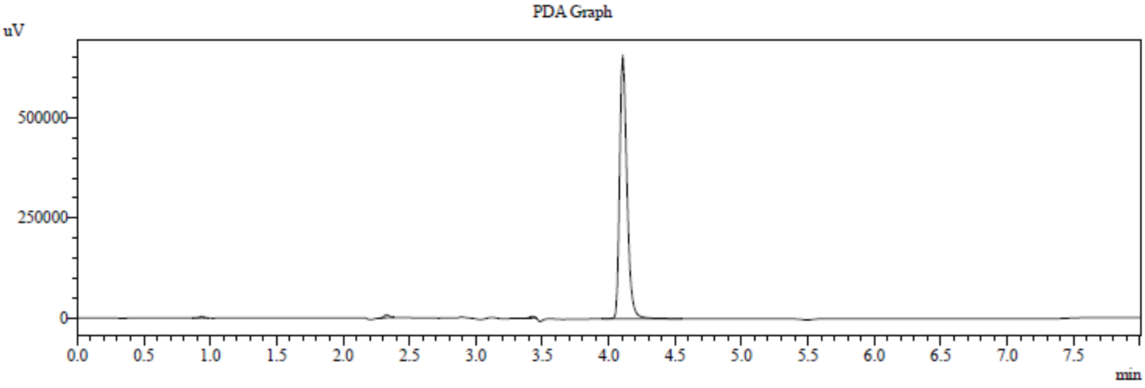

PDA Ch1 254nm 4nm

Peak#	Name	Ret. Time	Area	Area %
1		0.931	9586	0.375
2		2.322	20123	0.786
3		3.421	11318	0.442
4		4.101	2517955	98.397

MS Spectrum Graph

#:1 Ret.Time:Averaged 4.170-4.190(Scan#:418-420)
Mass Peaks:4 Base Peak:232.10(595337) Polarity:Pos Segment1 - Event1

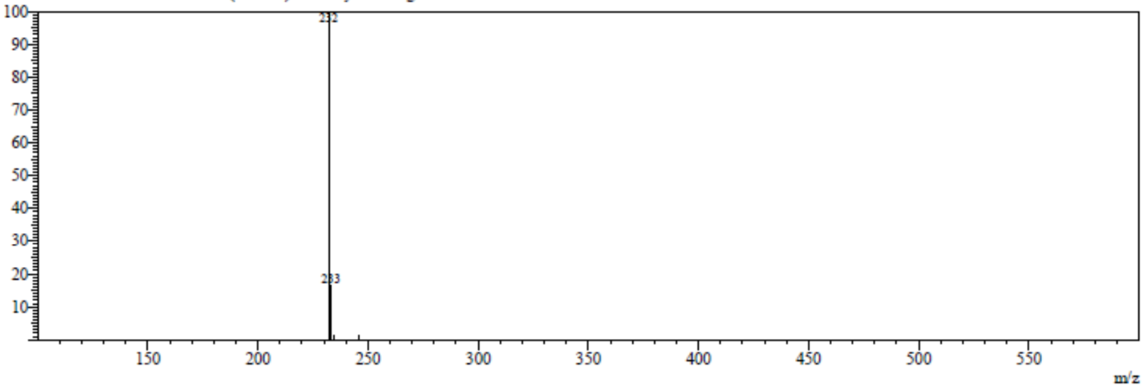

MS Spectrum Table

#:1 Ret.Time:
BG Mode:Calc 4.040<->4.590(405<->460)
Mass Peaks:4 Base Peak:232.10(595337) Polarity:Pos Segment1 - Event1

#	m/z	Abs.Inten.	Rel.Inten.	Charge	Polarity	Monoisotopic	#	m/z	Abs.Inten.	Rel.Inten.	Charge	Polarity	Monoisotopic
1	232.10	595337	100.00				3	234.20	7922	1.33			
2	233.10	97274	16.34				4	246.05	6498	1.09			

Figure S8. HPLC-MS chromatogram of compound **42**.

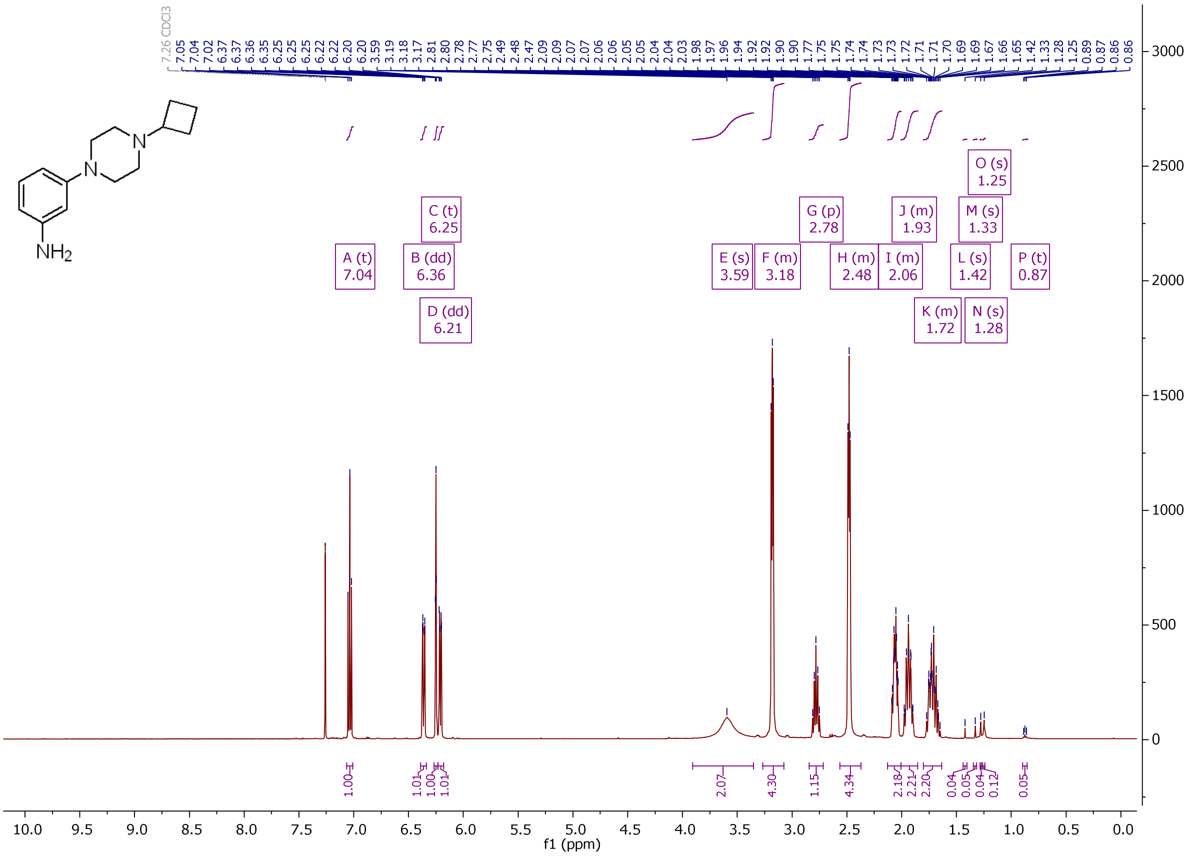

Figure S9. ^{1}H NMR spectrum of compound **42** in $CDCl_3$. Minor impurities (e.g. grease) are visible in the aliphatic range.

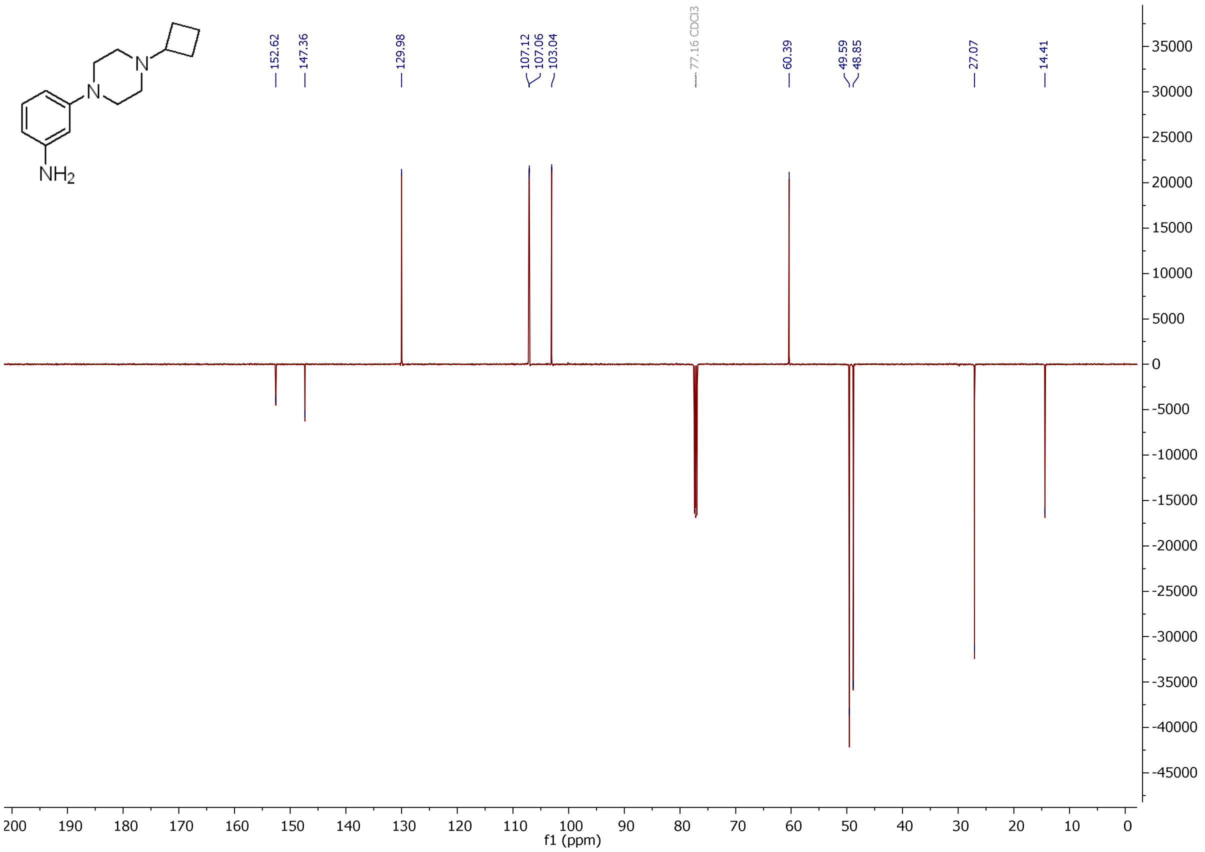

Figure S10. ^{13}C NMR spectrum of compound **42** in $CDCl_3$.

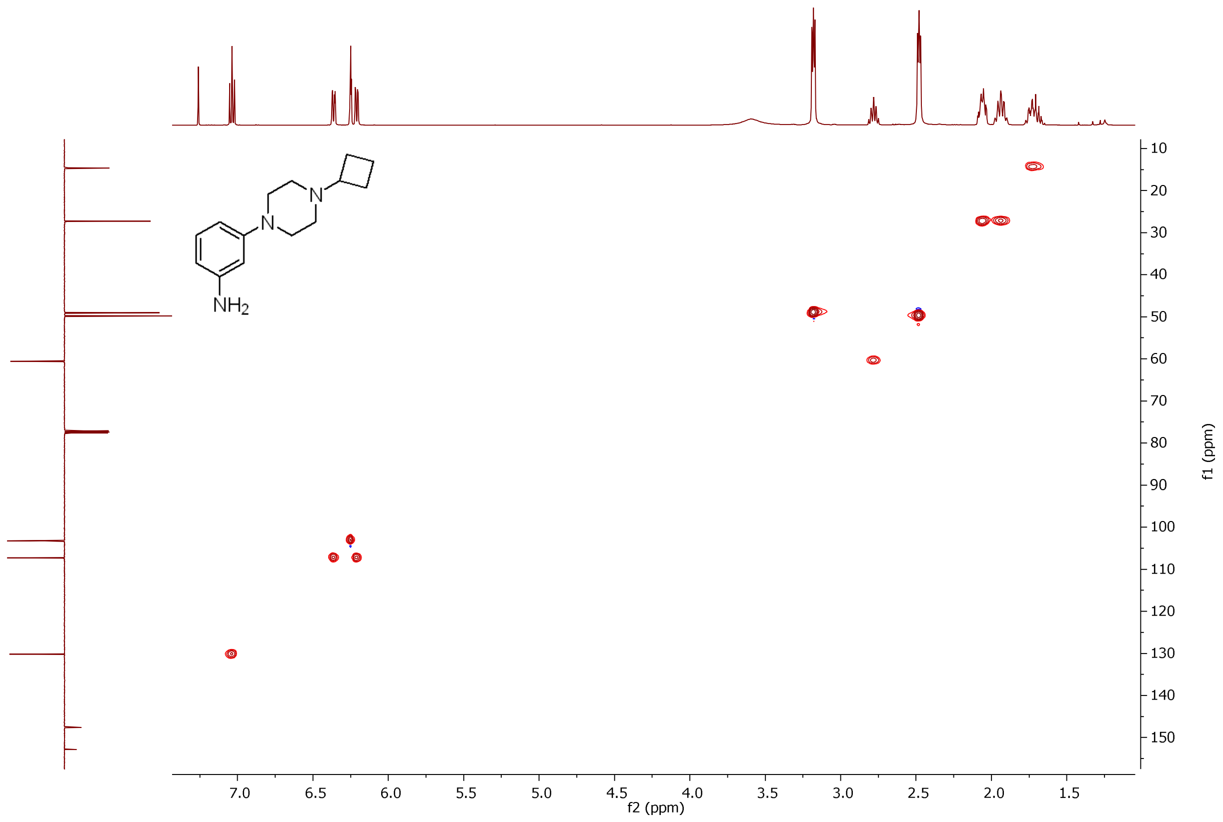

Figure S11. HSQC NMR spectrum of compound **42** in $CDCl_3$.

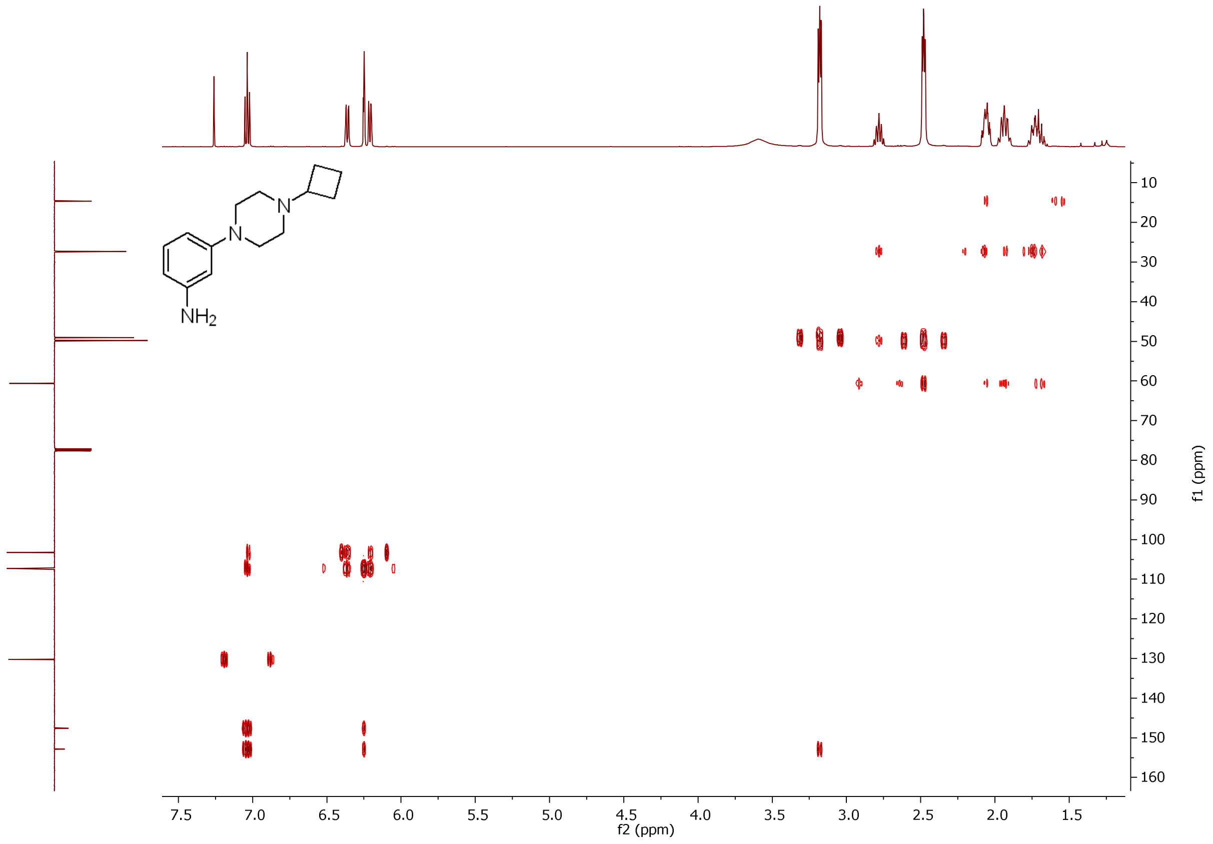

Figure S12. HMBC NMR spectrum of compound **42** in $CDCl_3$.

References

1. Mocking, T.A.M.; Verweij, E.W.E.; Vischer, H.F.; Leurs, R. Homogeneous, Real-Time NanoBRET Binding Assays for the Histamine H_3 and H_4 Receptors on Living Cells. *Mol. Pharmacol.* **2018**, *94*, 1371–1381.
